# Classification of the extracellular fields produced by activated neural structures

**DOI:** 10.1186/1475-925X-4-53

**Published:** 2005-09-07

**Authors:** Samantha Richerson, Mark Ingram, Danielle Perry, Mark M Stecker

**Affiliations:** 1 Department of Biomedical Engineering, Bucknell University, Lewisburg, Pa 17837 USA; 2 Department of Physics, Bucknell University, Lewisburg, Pa 17837 USA; 3 Department of Neurology, Geisinger Medical Center, 100 N Academy Rd, Danville, Pa 17822 USA

**Keywords:** Cable Properties, Dipole Moment, Quadrapole Moment, Action Potential

## Abstract

**Background:**

Classifying the types of extracellular potentials recorded when neural structures are activated is an important component in understanding nerve pathophysiology. Varying definitions and approaches to understanding the factors that influence the potentials recorded during neural activity have made this issue complex.

**Methods:**

In this article, many of the factors which influence the distribution of electric potential produced by a traveling action potential are discussed from a theoretical standpoint with illustrative simulations.

**Results:**

For an axon of arbitrary shape, it is shown that a quadrupolar potential is generated by action potentials traveling along a straight axon. However, a dipole moment is generated at any point where an axon bends or its diameter changes. Next, it is shown how asymmetric disturbances in the conductivity of the medium surrounding an axon produce dipolar potentials, even during propagation along a straight axon. Next, by studying the electric fields generated by a dipole source in an insulating cylinder, it is shown that in finite volume conductors, the extracellular potentials can be very different from those in infinite volume conductors. Finally, the effects of impulses propagating along axons with inhomogeneous cable properties are analyzed.

**Conclusion:**

Because of the well-defined factors affecting extracellular potentials, the vague terms far-field and near-field potentials should be abandoned in favor of more accurate descriptions of the potentials.

## Background

The most commonly employed neurophysiologic techniques used to diagnose and monitor the status of the nervous system in humans involve recording extracellular fields generated by time dependent changes in transmembrane potentials in axons, dendrites or cell bodies [1,2]. In order to understand these extracellular fields, it is important to classify the various types of field and their generators. Historically, evoked potentials were classified into "far-field" potentials and "near-field" potentials according to the criteria in Table 1[3-6]. These definitions are quite different from those employed in classical electromagnetic theory [7]. In electromagnetic theory, far-field potentials refer to the dominant component of the electromagnetic field in the range where the distance between the observation point and the generator is much larger than both the wavelength of the radiation and the size of the generator. The goals of this paper are to set forward expressions for extracellular fields generated under a number of circumstances and to use this information to produce a physically based classification scheme for the different types of extracellular field that may be recorded. The first step in this process will be a detailed description of the extracellular potentials generated as an impulse travels down a generalized neural structure. This will provide an understanding of those geometric properties of a generalized axon which are associated with the production of dipolar and quadrupolar fields at large distances from the axon. Following this, the effects that an inhomogeneous extracellular electrical environment has on the recorded extracellular field will be explored in a discussion of two problems: an impulse passing near a plane conducting boundary and an impulse passing near a conducting sphere. The effects of montage selection and low frequency filtering on these extracellular recordings will be investigated. For comparison, the effects that boundary conditions have on the distribution of extracellular fields will also be explored. Finally, the extracellular fields generated by an impulse traveling down an axon whose physical properties change abruptly will be investigated within the cable model. The time course of changes seen in the extracellular fields in the presence of inhomogeneities will lead to a detailed discussion of the frequency spectrum of extracellular potentials. In each of these cases, a theoretical discussion will be presented followed by the results of illustrative simulations.

**Table 1:** Classical Properties of Near and Far Field Potentials

Property	Far-Field	Near-Field
Latency	Relatively Independent of Recording Electrode Position	Strongly dependent on Position of Recording Electrode
Distribution on Skin	Broad	Narrow
Polarity	Positive	Negative or Positive
Recording	Monopolar	Monopolar or Bipolar

It should be noted that in many parts of the paper the terms charge, dipole, or quadrupole will be used although it is more proper to refer to point current sources, dipolar or quadrupolar current sources. This does not alter any of the fundamental conclusions in this paper.

### Model derivations and simulations

#### A. Extracellular fields and axonal geometry

Understanding how the geometry of a generalized axon affects the extracellular fields produced when it is depolarized is particularly instructive. Previous work by Plonsey and Rosenfalck was particularly useful in defining extracellular fields for active fibers of finite and infinite length in infinite homogenous media [8-10]. However, these models used previously assume fibers have circular cross sections, are straight, and are located in uniform conducting media. Holt and Koch [36] have studied this problem from the viewpoint of the cable equations. The general solution we present here is based on the geometry of surfaces, a simplified version of which has been applied previously in analyzing magnetic stimulation of a bent neuron [11,14]. Any surface, such as the surface of the generalized axon, can be characterized by a vector function of two variables [12]. In this development, these two variables will be called s and *θ*. It will be instructive to think of s as the distance along the length of the axon and *θ *as the angular position around the axon. The extracellular field generated when a small region of a nerve along its long axis is depolarized is due to relatively localized movements of charges. Thus, the extracellular fields in an infinite volume conductor can be calculated using the multipole expansion, the first terms of which are the field generated by the net dipole moment and the net quadrupole moment of the source. Appendix A (see Additional file #1) demonstrates the calculation of the net dipole and quadrupole moments produced by depolarization of the nerve in the case in which the trans membrane potential *V*_*m*_(*s*, *θ*) = *V*_*m*_(*s*) is independent of *θ*. This leads to the following expression for the dipole moment per unit activated length:



and the expression for the magnitude of the dipole moment per unit length:



In the above expressions,  and  are the unit tangent and normal vectors to the curve  describing the curve of the centroids of the axon and s is arc-length along this curve. κ*(*s*) is the curvature [12] of  at s. In addition:



where *a*(*s*, *θ*) is the distance from the centroid at s to the point (*s*, *θ*) on the surface. This is the mean square radius of the axon at position s.

One implication of the above equations is that the dipole moment produced by each segment of the axon depends only on the curvature of the curve of centroids and the change in the mean square radius with distance. It does not depend on the detailed shape of the axon. It is also important to realize that the component of the dipole moment produced by changing axonal radius is directed along the tangent to the centroid curve X*(*s*) and the component produced by the curvature of the axon are oriented along the normal to this same curve.

**Figure 1:**
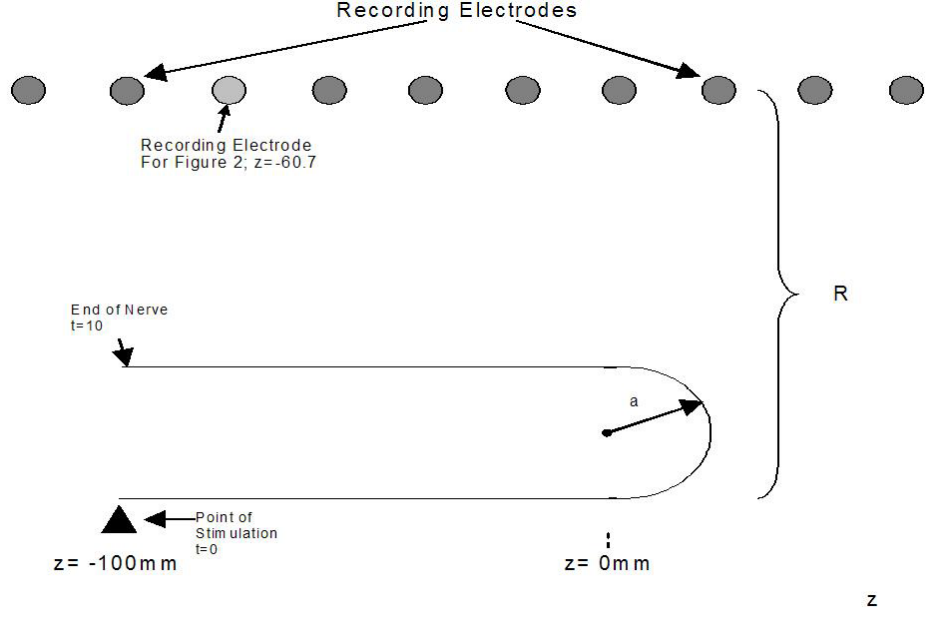
Illustration of the geometry used in simulating the extracellular fields generated when a nerve impulse propagates through a 180° bend with radius a = 60 mm. The separation between the leading and trailing dipoles is taken as 6 mm. The recording electrode position from which the tracings are taken is located at z = -60.7 mm. The leading and trailing dipoles [13] are oppositely oriented and parallel to the tangent of the nerve.

**Figure 2:**
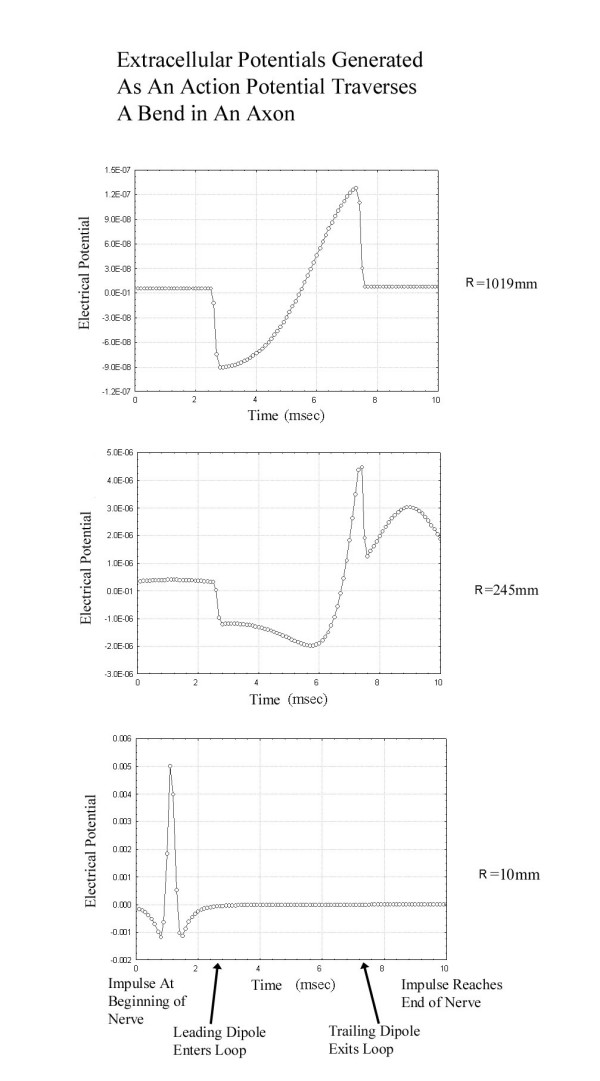
Tracings of the recorded extracellular potentials as a function of time. The tracing from the electrode closest to the axon (R = 10 mm) shows a typical quadrupolar traveling wave as the impulse passes closest to the recording electrode. As the recording electrode is placed progressively further from the axon (R = 245 mm and R = 1019 mm) the quadrupolar potential from the action potential passing near the electrode is dwarfed by the dipolar potential generated as the impulse travels through the bend. The impulse begins at t = 0 at z = -100 mm and by t = 10 reaches z = -100 again. The impulse reaches the location of the recording electrode at t = 1.1. The velocity of the travelling action potential was taken as 39 m/sec.

The quadrupole moment tensor produced when a small axonal segment is depolarized can be evaluated in a similar way although with substantially more algebra (See appendix A (see Additional file #1)) :



where τ*(*s*) is the torsion [12] of  and the  and  are integrals of the cube of the radius multiplied by sin(*θ*) and cos(*θ*) as noted in appendix A (see Additional file #1). This has a number of simple implications. First, whenever the axon cross section has inversion symmetry around the centroid (*a*(*s*, *θ*) = *a*(*s*, *θ *+ *π*)), then  and  are both zero and:



In order to illustrate these points, a simple simulation using the leading-trailing [13] dipole model was carried out to compute the expected extracellular fields generated as a function of time when a nerve impulse traverses a bend in an axon as shown in Figure 1. The leading and trailing dipoles are each oriented along the local tangent to the axon and are separated by a constant distance. Figure 2 shows an example of the extracellular fields demonstrating that when the recording electrodes are near the axon (small values of R) peaks are seen as the traveling impulse passes nearest the electrodes. However, far from the axon (large values of R), a peak is recorded at the point where the impulse traverses the bend. As expected, far from the source, the dipolar potential generated at the curve in the axon which declines as  for large distances dominates the quadrupolar potential which declines as .

#### B. Changes in local extracellular electrical environment

Once the effects of axon geometry on the recorded extracellular potentials have been explored, it is natural to explore the effects that changes in the extracellular electrical environment may have.

It has been suggested that the presence of inhomogeneities in the extracellular space can change the fundamental characteristics of the extracellular fields, producing "far-field" potentials (A discussion of the inverse problem of stimulating a nerve near a region with a localized change in conductivity can be found in Roth [14] and a discussion of the general effects of inhomogeneities in conductivity can be found in Geselowitz [15].). In order to understand these effects, it is helpful to begin by studying the fields generated by a small conducting sphere of radius a placed at position  in an arbitrary electric potential . An approximate expression for the potential produced by the presence of the sphere  far from the sphere is (see Appendix B (see Additional file #1)):



Therefore, as long as the electric field  that would have been present at the center of the sphere in its absence is non-zero, a dipolar potential can be generated by this conducting sphere. This is true even if that field is due to a quadrupole or higher order source so that the presence of inhomogeneities can produce extracellular potentials that decay much more slowly than those of the driving field. More generally, it is known [16] that the dipole moment induced in any dielectric (or conducting) object by an external electric field is:



where v is the volume of the object and  is a constant tensor that depends on the geometry of the object and the difference between the dielectric constant (or conductivity) of the object and the medium. For a sphere with dielectric constant (*ε*) (or conductivity (*σ*)) differing from that of the remainder of the environment:



where, *ε*_0 _is the dielectric constant of the rest of the medium and *δ*_*ij *_is the Kronecker delta. A similar expression holds for the case of a conducting sphere with *ε *replaced by *σ *and *ε*_0 _replaced by *σ*_0_, the conductivity of the medium outside the sphere.

In contrast to the results obtained above, it is important to note that dipolar fields are NOT generated when a nerve impulse characterized by a quadrupolar source travels near a plane conducting boundary. The effect of the plane boundary on the extracellular fields can be simulated by image charges placed at the location that their optical images would have in the boundary plane. Thus, if the source charge density is quadrupolar, the image charges are quadrupolar.

**Figure 3:**
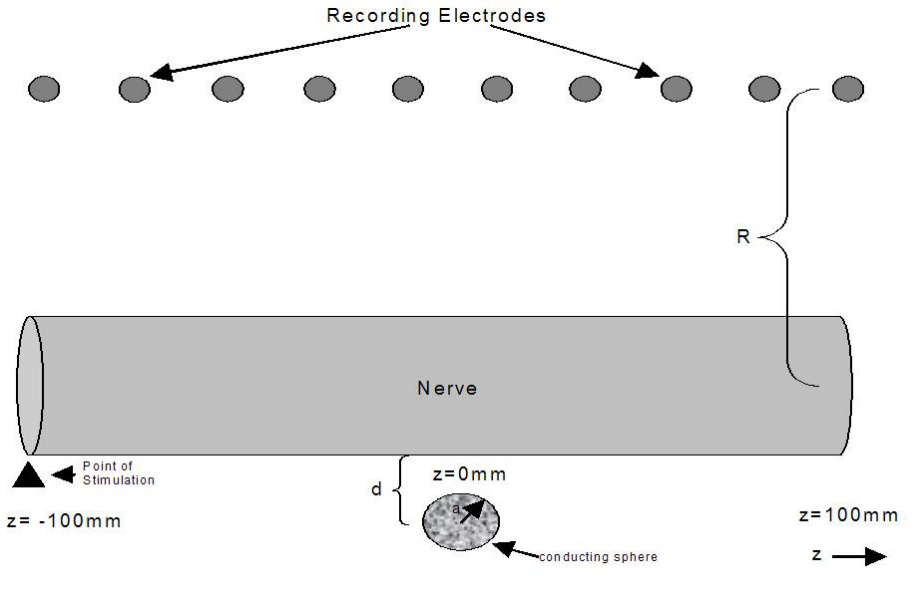
Illustration of the model used for the leading/trailing dipole simulation of the extracellular fields near a conducting sphere. R is the distance of the recording electrodes from the center of the axon. The distance between the leading and trailing dipoles is taken as 6 mm. The nerve is stimulated on the left side (negative z values) and propagates toward the right side. The sphere is taken as highly conducting with a radius of 1 mm and is placed 1.1 mm below the nerve. The center of the sphere is taken at z = 0.

**Figure 4:**
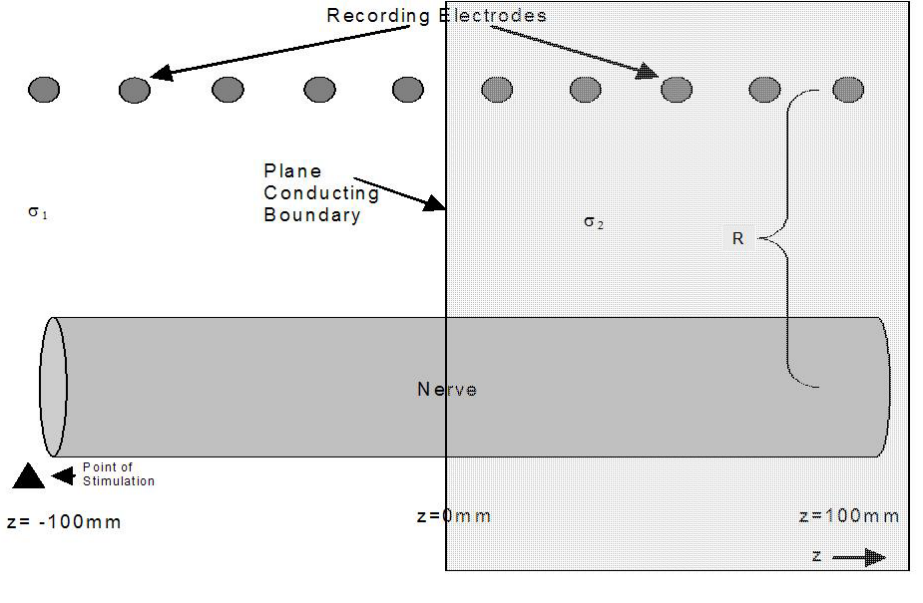
Illustration of the model used for the leading/trailing dipole simulation of the extracellular fields near a plane boundary. R is the distance of the recording electrodes from the center of the axon. The distance between the leading and trailing dipoles is taken as 6 mm. The nerve is stimulated on the left side (negative z values) and propagates toward the left side. The region of high conductivity is on the right z > 0 and the region of lower extracellular conductivity is on the left z < 0.

**Figure 5:**
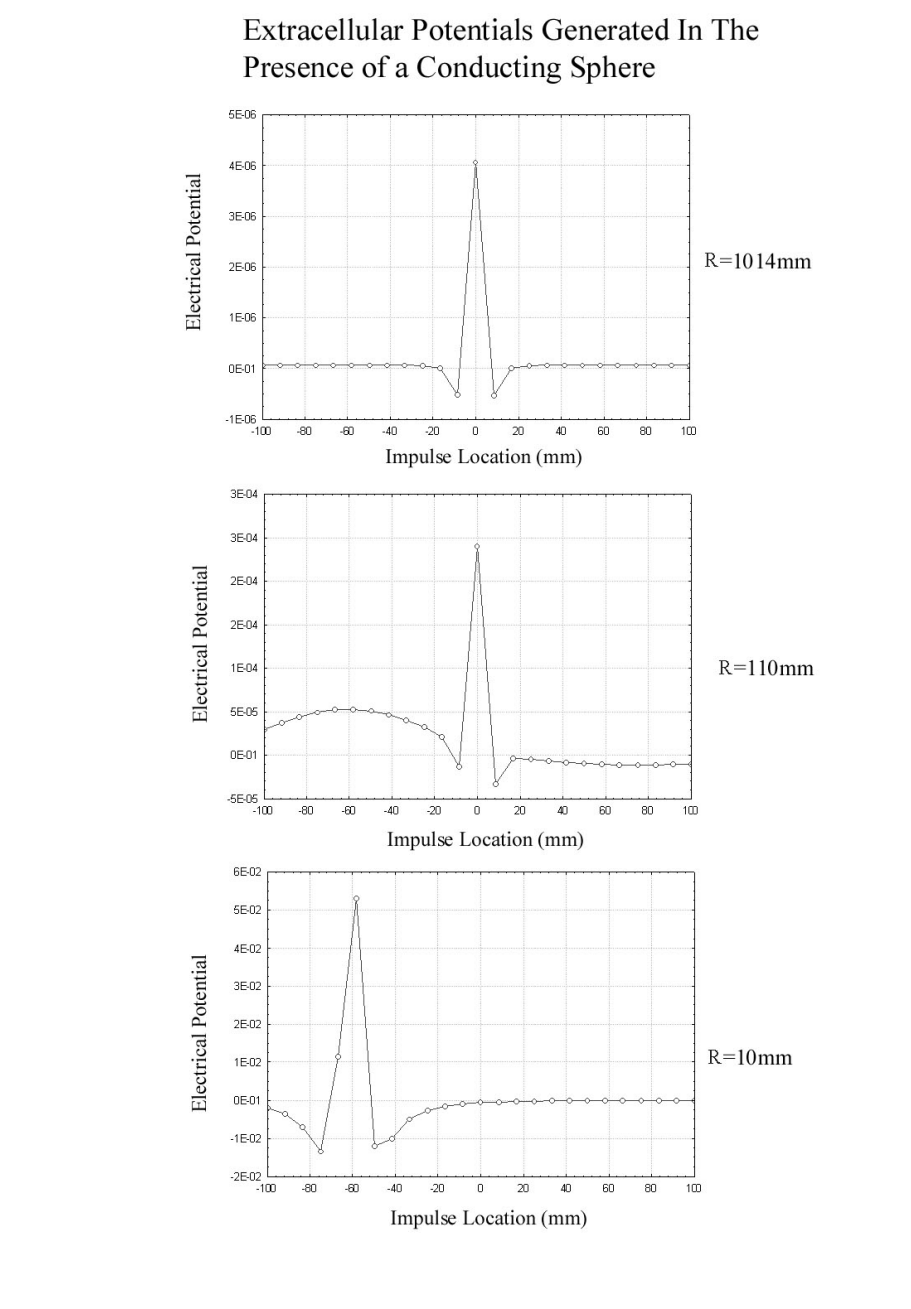
Plots of the extracellular potential recorded at an electrode a distance R from the axon located at z = -59 mm as the impulse travels from z = -100 mm to z = 100 mm. The geometry is that of the spherical conductor placed as in Figure 4. When the electrodes are located close to the axon (R = 10 mm), the peak in the potential occurs as the nerve impulse passes near the electrodes. For electrodes far from the axon (R = 110 mm or R = 1014 mm) the peak extracellular potential is recorded as the impulse travels nearest the sphere (z = 0). The values of the potential are arbitrary and are for purposes of comparison of the different graphs within this figure only.

It is instructive to compute the actual extracellular fields generated both when an impulse passes near a conducting sphere as in Figure 3 and near a conducting plane boundary as in Figure 4. These simulations were based on the leading/trailing dipole model with the leading and trailing dipoles oppositely oriented parallel and antiparallel to the z axis separated by a distance of 6 mm. The method of images [7] was used to compute the fields that satisfy the appropriate boundary conditions outside the sphere after breaking each dipole into closely spaced charges of opposite charge. Figure 5 shows the potential as a function of nerve impulse location in the case of the conducting sphere for electrodes near and far from the axon. It is clear that when the recording electrodes are near the axon (R small) the peak in the potential is seen as the impulse traverses the point nearest the recording electrodes while, when the electrode is further from the axon (large R), the peak in the extracellular potential appears as the impulse passes nearest to the conducting sphere. This is what would be expected on the basis of the dipolar potential generated as the impulse approaches the sphere.

**Figure 6:**
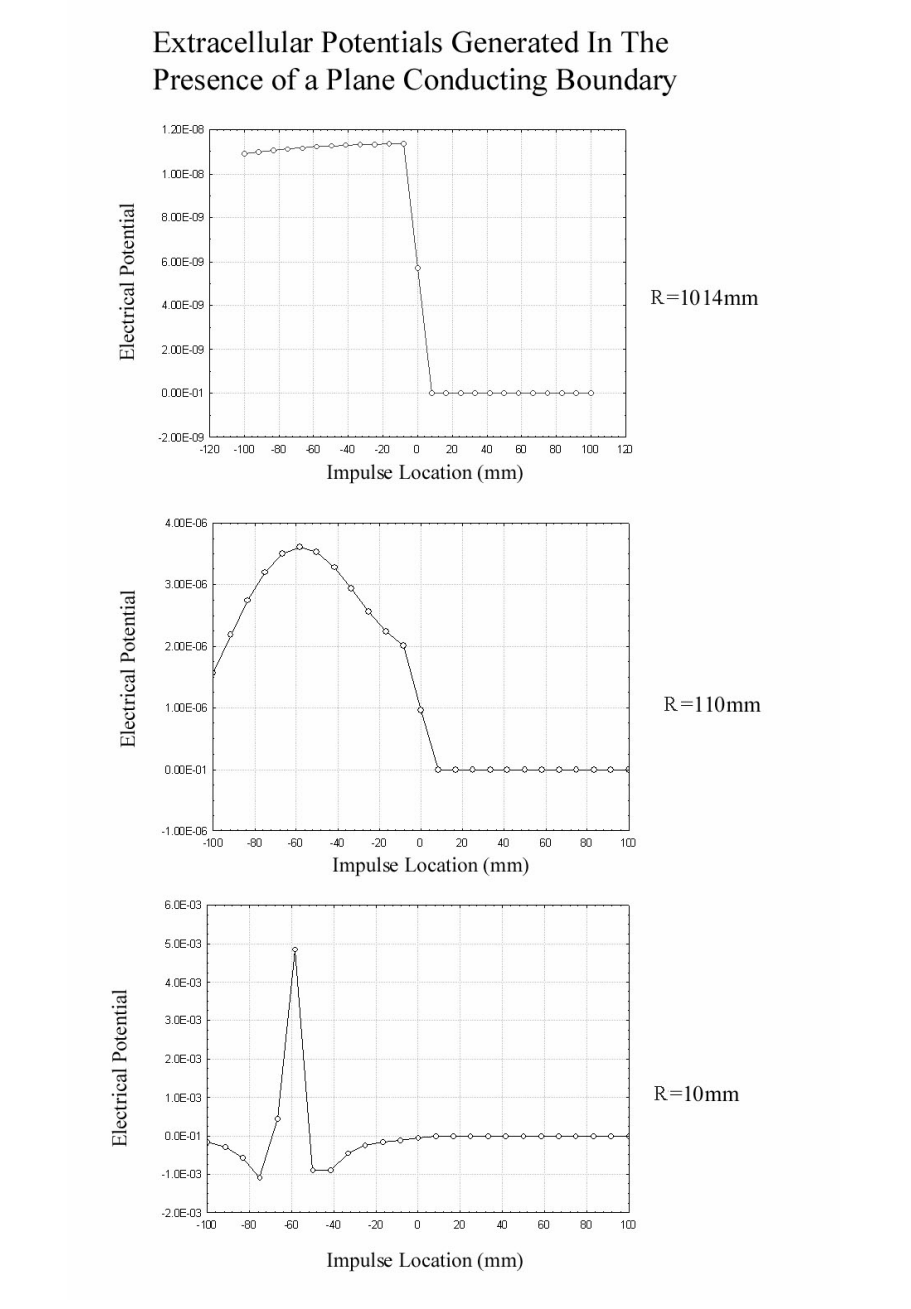
Plots of the extracellular potential recorded at an electrode (located at z = -59 mm) a distance R from the axon as the impulse travels from z = -100 mm to z = 100 mm. The geometry is that of the plane conducting boundary placed as in Figure 5. When the electrodes are located close to the axon (R = 10 mm), the peak in the potential occurs as the nerve impulse passes near the electrodes. For electrodes far from the axon (R = 1014 mm) a step change in potential is seen as the impulse passes through the boundary between the two regions. The values of the potential are arbitrary and are for purposes of comparison of the different graphs within this figure only.

Figure 6 shows the extracellular fields produced as an impulse passes through a plane conducting boundary. It is clear that the presence of the plane conducting boundary is not associated with a peak in the extracellular field as the impulse traverses the boundary as in the case of the impulse near the conducting sphere. Again, this has its origin in the fact that the potential drops as  at large distances from the axon in the case of the conducting sphere and  for the plane conducting boundary.

#### C. Effects of finite volume conductors

Since practical recordings are not carried out in an infinite volume conductor, it is useful to consider a simple qualitative model demonstrating the effects that placing a charge in a finite volume conductor can have on the recorded extracellular fields (Appendix C (see Additional file #1)). In particular, the special case of charges placed inside a cylindrical volume conductor that is insulating except at its two ends is very instructive. Assuming that all charges are confined to a finite segment of the cylinder z_0 _- Δ <*z *<*z*_0 _+ Δ, the potential outside the region in which the charges exist can be written:



It is instructive to consider the mean value of the potential along segments perpendicular to the long axis of the cylinder:



These integrals are not well defined in an infinite volume and so the results are restricted to finite cylindrical volume conductors. Integrating the Poisson equation over the radial and angular variables yields an expression for the mean potentials in the charge free regions and integrating the Poisson equation across the region containing charges produces the following expression for the averaged electrical potential:



In this equation, Q is the total charge contained in the region and d_z _is the component of the dipole moment of the charge along the long axis of the cylinder. *a*_<_and *b*_<_are constants chosen to satisfy the boundary conditions. This suggests that, far enough from the sources, the actual potential has a linear dependence on the axial coordinate. The form of the above equation suggests that if the source is dipolar there may be a step change in the potential across the region containing the charges that is proportional to the dipole moment. A quadrupolar field would be expected to be least influenced by the presence of the finite cylindrical volume conductor. These results are similar to those obtainable using the Green's function for the appropriate cylindrical boundary conditions [17].

The critical question is to define the regime in which these results apply. Some arguments relating to this question are presented in Appendix C (see Additional file #1). They suggest, for an infinitely long cylinder, that when the axial coordinate of the point of observation is many cylinder diameters from the charges (*z *>> *a*), the potentials develop the linear behavior discussed above. Similarly, when the point of observation is much less than a cylinder diameter away from the charge, it is expected that the potential will possess similarities to that seen in an infinite volume conductor.

**Figure 7:**
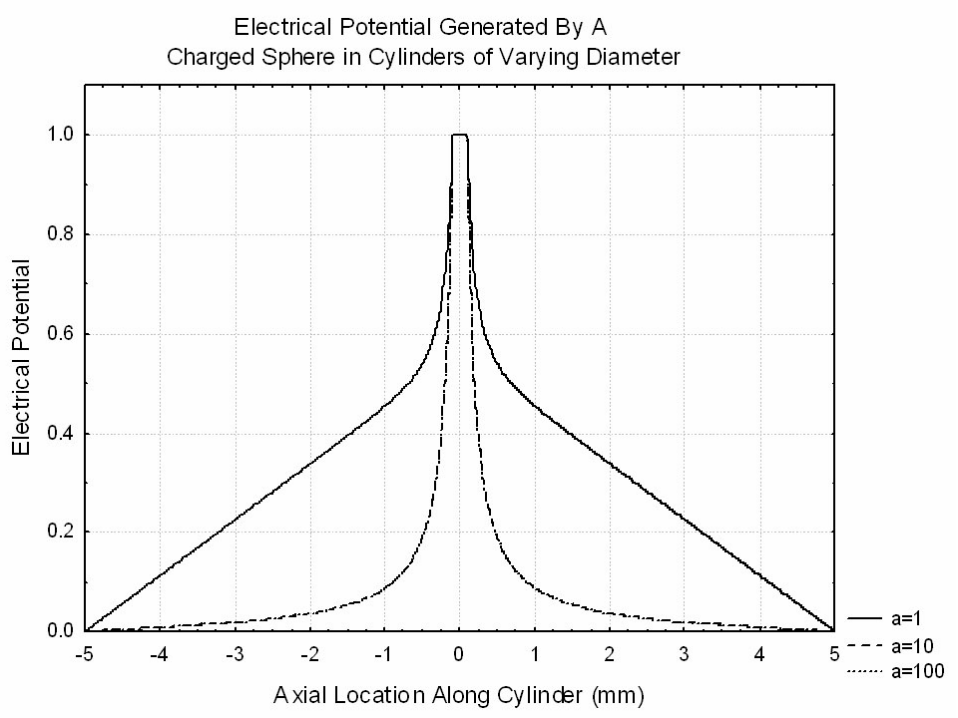
The potentials generated by a conducting sphere of radius 0.1 mm held at constant potential 1 placed in cylindrical volume conductors of differing sizes. The flat ends of the cylinder are at ground potential and the curved surface is taken as an insulator. Note that the cylinder has length 10 mm and extends from -5 to 5 on either side of the point z = 0.

**Figure 8:**
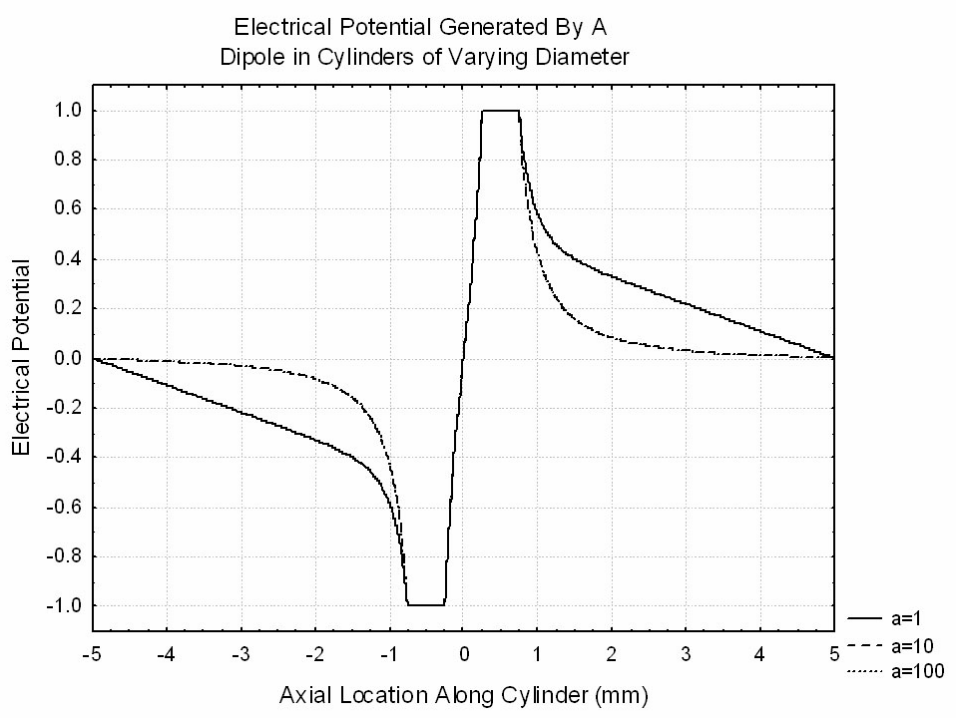
The potentials generated by a "dipole" formed from two conducting spheres of radius 0.25 mm centered around z = +/-0.5 mm held at constant potential 1 and -1 respectively placed in cylindrical volume conductors of differing sizes. The flat ends of the cylinder are at ground potential and the curved surface is taken as an insulator.

**Figure 9:**
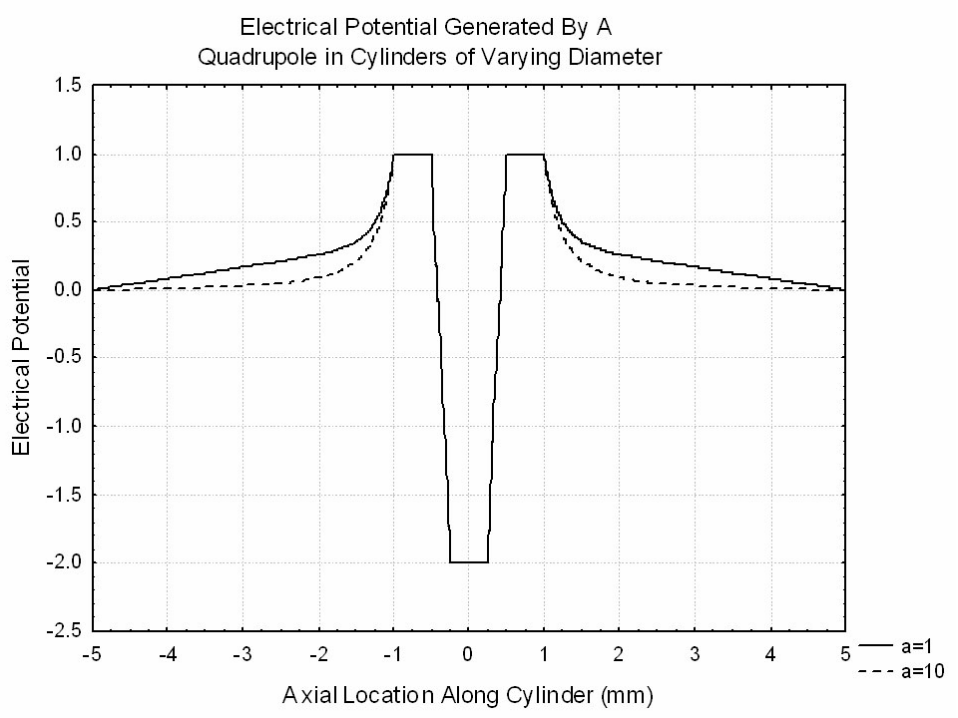
The potentials generated by a "quadrupole" formed from three conducting spheres of radius 0.25 mm centered around z = +/-0.75 mm and z = 0 held at constant potential 1,1,-2 respectively placed in cylindrical volume conductors of differing sizes. The flat ends of the cylinder are at ground potential and the curved surface is taken as an insulator.

In order to understand the effects of finite volume conductors in more detail, a series of finite element simulations were performed using FEMLAB (Comsol, Natick MA). In each of these simulations the Laplace equation was solved using an algebraic preconditioner followed by a conjugate gradients equation solver. Results were checked for stability to changes in grid sizes. In order to allow for reasonable solutions with manageable grid sizes, finite sized spheres were used in place of point charges. Figure 7 illustrates the distribution of potential from a conducting sphere of size 0.1 held at a potential of unity placed in the center of a cylinder of length 10 and radii ranging from 1 to 100. The ends of the cylinder were grounded and the remainder of the cylinder was considered to be insulating. This clearly demonstrates that at roughly half a cylinder radius from the sphere, the potentials deviate from the values that they would have in an infinite volume conductor and demonstrate linear changes with z. Figure 8 demonstrates the potential produced by a dipole simulated by two conducting spheres of diameter 0.25 at +/-0.5 are held at potentials of +/-1. Smaller spheres and smaller spacing between spheres required prohibitively large grids for solution. It is clear that the transition to the linear behavior discussed above occurs only for the thin cylinder. Finally, Figure 9 shows the potential produced by a quadrupole simulated by three spheres of diameter 0.25 centered at 0.75, 0, -0.75 with charges 1,-2,1. It is clear that the finite volume conductor has much less effect on the potential generated by the quadrupole than those generated by the dipole or the monopole as expected.

**Figure 10:**
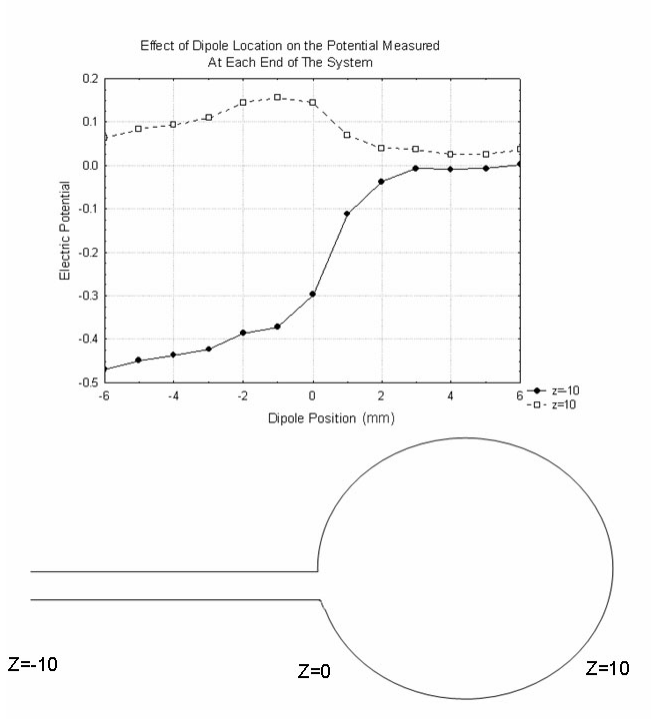
The effects of more complex geometries of the volume conductor on the electric potentials recorded. All results refer to the following geometry. A fully insulated cylinder of radius 1 and length 10 extending from z = -10 to z = 0. This cylinder is attached to an insulated sphere of radius 5 with the two intersecting at z = 0. The plots show the potential at the flat cylindrical end (z = -10) and the end of the z = 10 spherical edge as a function of the axial location of the center of a dipole consisting of two spheres held at potentials of 1 and -1 as in Figure 8. The step in the voltage at the cylindrical end of the volume as the dipole moves through the boundary between the two regions is evident.

Figure 10 further illustrates the properties of the volume conductor that affect the extracellular fields. It shows the results of finite element simulations for a case in which a thin (radius = 1, length = 10) insulating cylinder is attached to a larger (radius = 5) insulating sphere as would be the case if an arm were attached to a larger torso. Specifically this figure demonstrates the potential recorded from the z = -10 end of the cylinder and the z = +10 end of the sphere for a dipole source placed at different locations along its long axis. As expected, the potentials at the ends of the cylindrical region exhibits a step change as the dipole passes from the cylindrical end of the figure into the spherical end. The behavior of the potential in the spherical region is more complex.

The sensitivity of the recorded extracellular potentials to the shape, size and character of the volume conductor in which the sources are immersed is evident in these calculations. They are also very sensitive to the detailed structure of the boundary conditions imposed. These observations are consistent with the results obtained in a number of experiments performed by Jewett [6,18] who recorded changes in extracellular potentials from isolated nerves as the impulse propagated into volume conductors of different sizes and shapes. These observations on the importance of finite volume effects are also consistent with the conclusions drawn from finite element models of Cunningham [19] who evaluated extracellular fields created in a 2-D finite element model of the hand, arm and torso. However, as the geometry of the system becomes more complex, the interpretation of results also becomes more complex. Mapping studies of the electric fields generated by artificial dipole and quadrupole sources by Dumitru and King [20,21] reproduce the above theoretical and computational results and in particular demonstrate that dipole sources in cylinders are associated with large regions of constant potential away from the source. However, these authors state that quadrupolar sources do produce fields far from the source when inside a cylinder under certain circumstances. This may result from the fact that in these simulations discrete electrodes were used to generate the quadrupolar source.

Clinical studies [22-24] on the distribution of the potentials generated by median nerve stimulation in humans do demonstrate some potentials with a latency that is independent of electrode position that are broadly distributed over the skin. The spatial distribution of these potentials does not appear to be quadrupolar or bipolar in nature as they seem to decrease in amplitude very slowly with distance from the putative source. It is thus likely that boundary effects play a significant role in the generation of these potentials.

#### D. Changes in cable properties of an axon

The effects that a localized alteration in the cable properties of an axon has on the generated extracellular fields are also of interest [25]. As long as the axon remains cable-like, straight and with a constant radius, equation (2) shows that only quadrupolar potentials will be generated even if cable properties of the axon change along its length. However, as in the case of the impulse approaching the plane boundary, the effective quadrupole moment will change near the point at which the cable properties change.

In order to illustrate these effects, a simple simulation was undertaken to find the transmembrane potential as a function of time for the situation in which a stimulator moving with a constant velocity injects a constant amount of current through the membrane. It is assumed that the membrane resistance quadruples at the point z = 100. The equations describing this simple model and the method of computing the extracellular fields is discussed in Appendix D (see Additional file #1).

**Figure 11:**
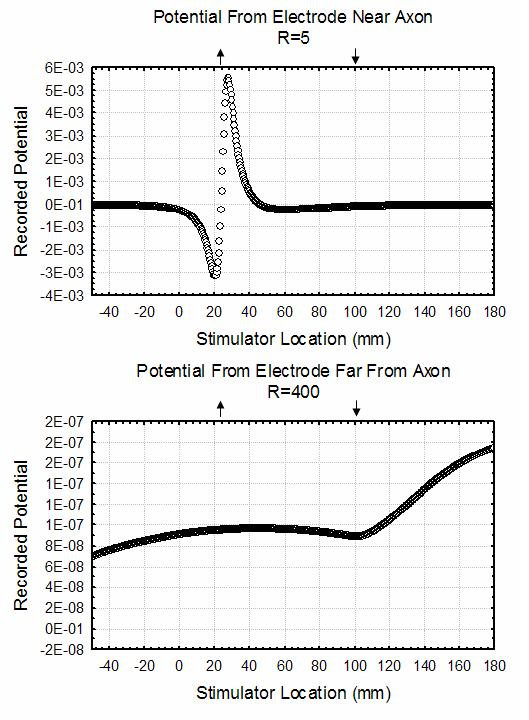
Plot of the potential generated at various distances from the stimulated non-uniform axon described equations (D.1) and (D.2) as a function of time for electrodes with varying distances from the axon. The recording electrode is located at the point z = 25 mm. In this graph, the abscissa is the location of the stimulator as it moves along the axon. The downward arrow denotes the location (z = 100 mm) where the space constant of the axon suddenly doubles and the upward arrow denotes the location (z = 25 mm) of the recording electrode. Note that for recording electrodes near the axon only the traveling wave is noted but when the electrodes are placed far from the axon, only potentials generated as the impulse reaches the boundary are evident.

Figure 11 contains a plot of the recorded potential at z = 25 as the impulse passes by this location as a function of the distance of the recording electrodes from the axon. This clearly demonstrates that, when the recording electrodes are close to the axon, only the traveling wave is recorded as the impulse passes near the axon. Far from the axon, much of what is seen in the figure is apparently generated as the impulse reaches the interface between the two regions of altered membrane resistance. In fact, much of this is a function of the time scale used to plot the data. As will become apparent in the next section, the frequency of propagating extracellular potentials recorded declines as the point of observation moves further from the axon. Thus, far enough from the axon, the propagating potential will be difficult to visualize although the potentials that occur as the impulse encounters a boundary having higher frequency components are easier to see. A detailed analysis of the potential seen at the time the impulse reaches the boundary supports this interpretation since it does fall as  at large distances from the axon and is quadrupolar.

#### F. Spectral analysis of extracellular potentials

As in the above section, it is clear that the frequency of the extracellular potentials produced as an action potential moves through a complex medium is an important factor in interpreting recordings. Thus, an understanding of the spectral content of the potentials recorded from a travelling action potential is important. This problem is discussed in detail in Appendix E (see Additional file #1) where it is shown that the power spectrum  at angular frequency *ω *of the potential recorded a distance R from an axon along which an action potential propagates at a constant velocity v is given by:



where *α *is the quadrupole moment associated with the action potential, *β *is the spatial extent of the action potential and:



It is important to note that (12) implies that, the spectral response is the product of two factors, a "form factor" related to the spectral content of the impulse itself  and a "structure factor"  related to the distribution of the potential from a quadrupolar source. In Appendix E (see Additional file #1) it is shown that 90% of the contribution of the "structure factor" to the total power in the recorded extracellular potential occurs over the frequency range:



Table 2 contains a list of the frequencies that correspond to these values for different recording electrode to axon distances, R, for an impulse travelling at a velocity of 40 m/sec. The critical observation is that higher frequencies are recorded near the axon and only low frequencies far from the axon. On the other hand, contributions from the "form factor" are independent of R but do depend on the spatial extent of the action potential. Ninety percent of the power in the "form factor" occurs for:

**Table 2:** Cutoff/Peak Frequencies For Action Potentials

R	*f*_max _(Hz)		*f*_*peak *_(Hz)
0.1 cm	74,000	1,262	1,273
1 cm	7,400	1,262	716
10 cm	740	1,262	95.4
100 cm	74	1,262	9.54



**Figure 12:**
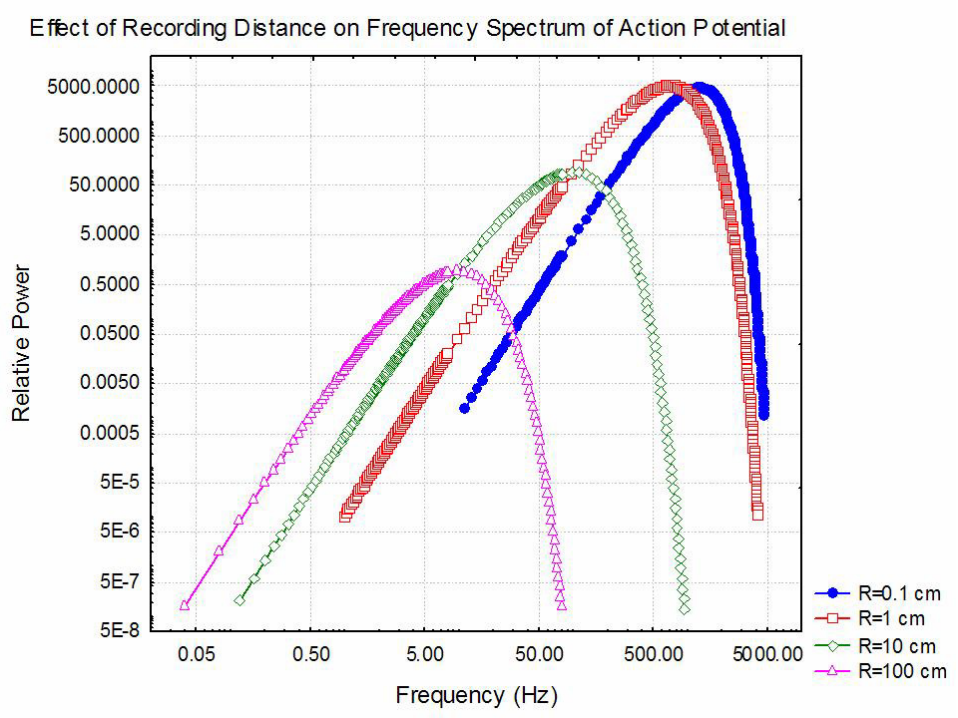
Plot of the power spectrum of extracellular fields generated by an action potential propagating along an axon as a function of distance, R, from the axon. It is assumed that the potential moves with a velocity of 40 meters/second and that the length of activated neuron is 6 mm. The maximum frequency for which there is significant power clearly declines as the distance between the axon and the recording electrode increases.

Taking *β *= 6 mm and v = 40 meters/second, it is possible to tabulate the cutoff frequencies for the structure and form factors as well as the location of the peak of the power spectrum as in Table 2. It is clear, as expected, that close to the axon, the highest frequency is determined by the shape of the action potential but, at large distances, the frequency content of the recorded action potential is more strongly determined by the distance from the axon. This is confirmed in Figure 12 which demonstrates the power spectra expected from (12) as a function of distance from the axon.

This argument demonstrates that applying a low frequency filter when recording electrodes are placed far from the source can remove the responses generated as the action potential traverses the axon. However, the frequency spectrum of the potentials generated when an impulse reaches any boundary must be similar to that of the "form factor" only since these potentials do not propagate down the axon (The "structure factor" component arises only out of propagation of the impulse as is evident since only this term contains the propagation velocity.). Thus, as in Table 2, if the distance between the recording electrodes and the axon is sufficiently great, a low frequency filter may eliminate the propagating potentials to a much greater degree than the potentials generated at interfaces. This may obscure the true nature of the generators of the recorded extracellular fields.

#### G. The effect of recording montage

**Figure 13:**
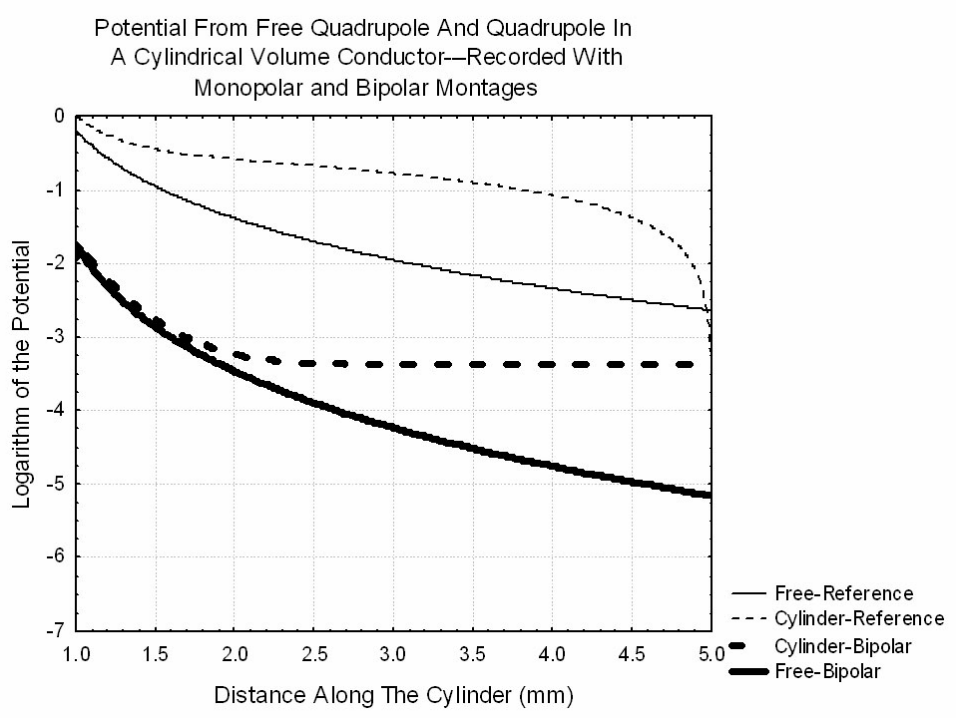
Plot of the potentials recorded from the quadrupole of Figure 9 in an infinite volume conductor and an insulating cylindrical volume conductor of radius 1 extending from z = -5 to z = 5. Recordings made with a monopolar electrode (reference recording) and a bipolar recording are shown. The logarithm of the absolute value of the potential is plotted rather than the potential in order to better show the behavior of the potential far from the source. Note that plots extend only from z = 1 to z = 5 although the cylinder extends from -5 to 5. This demonstrates that bipolar recordings far from the source yield a constant value independent of electrode location.

**Figure 14:**
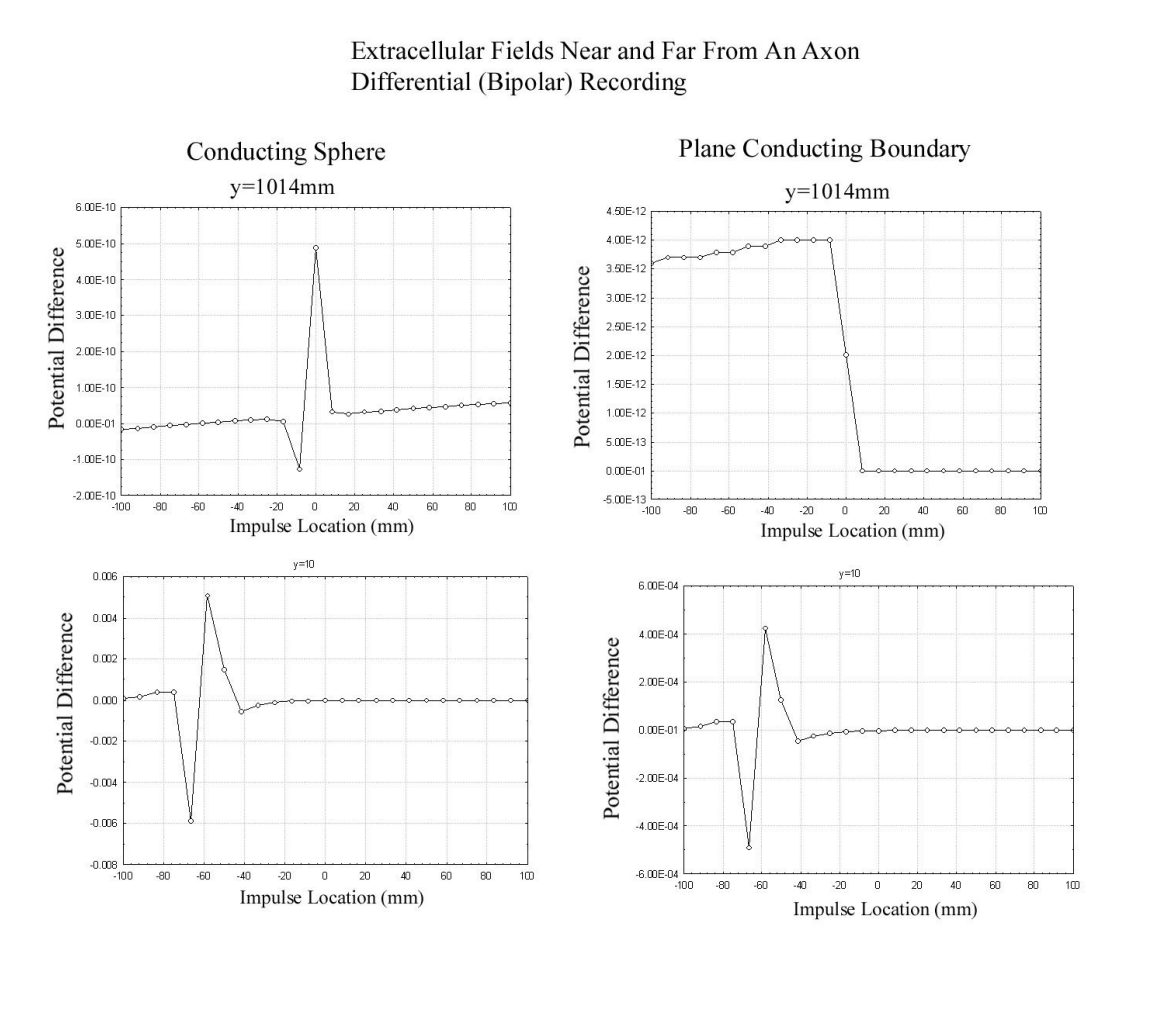
Plots of the potentials recorded in the same simulations as in Figures 5 and 6 using a bipolar montage with the two electrodes placed a short distance from one another in the z direction. This demonstrates essentially similar behavior to that seen with the monopolar recording.

It should be noted that all of the simulated extracellular potentials discussed above represent the absolute value of the potential at the recording point. This corresponds to the use of a referential recording montage. In many clinical situations, bipolar recordings of nerve action potentials are made in which the difference in potential between two closely placed electrodes is recorded. The question is whether there is any theoretical disadvantage to either of these recording techniques for certain types of extracellular fields. Figure 13 shows the logarithm of the absolute value of the potential produced by a quadrupole in an infinite volume conductor and in a cylindrical volume conductor computed as in section C above. The thin lines refer to reference recordings and the thicker lines refer to bipolar recordings. As suggested by Stegeman, both the bipolar and reference montages record potentials that vary with the location of the electrode when recordings are made in an infinite volume conductor while the bipolar recording in the cylindrical volume conductor produces only constant values when the electrode is more than 2 cylinder radii from the quadrupole. Thus, if recordings of a propagating action potential are important in a finite volume conductor, then reference recordings would be required. If only the changes that appear as a potential reaches some inhomogeneity then a bipolar montage would be adequate as seen by comparing the results of Figure 14 to those of Figures 5 and 6.

#### H. Radiation fields

Although electromagnetic radiation is not typically considered in clinical applications, changes in charge distributions over time produce radiation fields that typically decline in amplitude as  for large distances from the source and so could be significant when the recording electrodes are very far from the source. However, the detailed considerations of appendix F (see Additional file #1) indicate that these fields are miniscule in all potential situations.

## Discussion

In this paper, a number of theoretical mechanisms underlying the generation and recording of extracellular potentials during propagation of a nerve impulse down a generalized neural structure were presented and illustrated with data from simulations. This approach provides a much greater degree of insight into the generation of extracellular fields than the use of simple analytic approaches, finite element simulations, or clinical recordings in isolation. In particular, although necessarily approximate and idealized, the theoretical calculations allow for predictions of the general categories of field types that can be encountered and estimates of their magnitude.

When a neural membrane is depolarized the change in transmembrane potential is associated with a dipole moment density oriented perpendicular to the surface of the membrane that is proportional to the change in potential. Because of the symmetry of the ideal cylindrical axon, the net dipole moment associated with the activation disappears and so the propagating nerve impulse is typically a quadrupolar field far from the axon. However, when the cylindrical symmetry is broken by bending the nerve, a net dipole moment along the normal to the axon may be generated. In addition, if there are changes in the axon radius along the length of the cylinder there will be dipole moments generated directed along the tangent to the axon.

The theoretical results and simulations discussed above are in agreement with the experimental results obtained by Dupree and Jewett [26] which showed a peak in the extracellular potential that is generated when frog sciatic nerve was bent. These recordings were however made with electrodes always within 200 mm of an axon placed in a finite volume conductor. Because of the relative proximity of the electrodes to the source the full transition from recording only traveling waves to recording only potentials as the impulse enters the bend is not seen in this experiment.

In addition, the above theoretical results are applicable to situations in which an axon terminates in a sealed end. This is equivalent to allowing the axon radius to change from its baseline value to zero at the end of the axon. As described by (2) a net dipole moment will appear at such points and a peak will be recorded in the extracellular potentials. Equation (2) does not suggest that a net dipole moment will appear in a cylindrical axon with constant radius that is cleanly cut (ie not sealed) at the end. Dumitru and Jewett [13] suggest that this should occur because, once the impulse reaches the end of the axon, there is no longer "neural tissue to support the leading dipole". They speculate that at this point only the fields generated by the trailing dipole appear. This is not the case, since the leading and trailing dipoles are not physical entities and are only used to represent the field generated by the depolarized membrane surface. In fact, as the impulse reaches the cut end, the equivalent leading dipole must remain at the end of the axon while the trailing dipole gradually reaches the end of the axon. Thus, the quadrupole moment of the impulse will decline linearly to zero as the impulse reaches the cut end of the nerve since the total area of depolarization will diminish gradually. Thus, no peak in extracellular potential should be recorded as the impulse reaches a pure "cut end". However, if there is any change in axon diameter at the end, a large potential may be generated.

As breaking the cylindrical symmetry of an axon by distorting the axon itself can produce net dipolar fields, so can placing an axon in an environment that is not cylindrically symmetric. In particular, placing a sphere of altered conductivity near the axon destroys cylindrical symmetry and results in a dipolar potential even when extracellular fields generated by the axon itself would be quadrupolar. Placing an infinite conducting plane perpendicular to the path of the traveling impulse, however, does not destroy the symmetry of the depolarized axonal segments and so, although the effective quadrupole moment of the propagating action potential changes as the impulse passes through this barrier, no dipolar potentials are generated. Similarly, when a propagating impulse reaches a point on the axon at which there is a sudden change in the cable properties, there is a change in the quadrupole moment but no dipole potential is generated.

The results obtained in this section should be compared to the experiments of Nakanishi [27] who placed nerves through multiple partitions and found that, with one electrode in each partitioned segment, potentials were generated that could be related to the passage of the impulse from one compartment into another. The amplitude of this potential was correlated with the impedance between the partitions, a measure of the size of the gap in the partition. This also suggested that the geometry of the partitions was critical to the development of the responses. This seems plausible and recordings with electrodes either far from the axon or in multiple locations to detect the angular dependence of the potential could be helpful in determining whether the true character of the responses are dipolar or quadrupolar.

The above simulations of the potentials produced as an impulse passes through a plane conducting barrier are different from those found by Stegeman [28,29] who computed the potentials produced by a simulated impulse traveling in an insulating cylindrical shell filled with media of different conductivity. In this model, an action potential travels at constant velocity along the long axis (z axis) of the cylinder at its center and encounters a stepwise change in conductivity at the point z = 0. Their simulations suggested that, as the nerve impulse passes the point z = 0, it generates a stepwise constant shift in the electric potential at all points z > 0 although no or minimal change is seen for in the region of space z < 0. Although there are sudden changes in the potential in model described above as the impulse passes through the boundary between the regions of differing conductivity, all of the potentials do decline with distance from the source as would be expected from a quadrupolar source. This difference between Stegeman's model and the model of an action potential approaching a plane conducting barrier as discussed above relates to the unusual types of electric field that are generated in a finite cylindrical volume conductor. As discussed in section C, when sources are placed in an insulating cylinder, the recorded potential is similar in many ways to its value in an infinite volume conductor when the recording electrodes are very close to the source. However, neither dipolar or quadrupolar fields are recorded more than 2 or 3 cylinder radii from the source but the potential becomes a linear function of the axial coordinate. This behavior is specific to insulating cylindrical volume conductors. Although there are distortions of the potentials in a circular volume conductor and in a cylindrical volume conductor that is not perfectly insulating, many of the characteristics of the potentials recorded in an infinite volume conductor persist. Field distributions produced primarily as a result of boundary effects can be identified in actual recordings when the potential does not drop as  or  or when the expected angular behavior expected with a dipolar source, cos(*θ*), or a quadrupolar source, 3 cos^2^(*θ*)-1, are not evident.

The effect of recording montage on the recorded potentials was discussed. In any recordings of extracellular potentials in an infinite volume conductor, similar information is recorded from closely spaced electrodes and with a distant reference because the potentials drop off with distance in a predictable manner. However, the potentials generated by sources within a finite volume conductor with insulating boundary conditions become linear functions of distance far from the source and so bipolar recordings will not record changes in the location of the sources over time while monopolar or reference recordings will.

The spectral properties of the generated extracellular fields were also elucidated with the result that the frequency spectrum generated by a traveling impulse is cutoff at a frequency which is inversely proportional to the distance of the recording electrodes from the axon. Put differently, the frequency of potentials recorded far from a traveling quadrupolar action potential drops as the distance between the recording electrodes and the axon increases. On the other hand, the frequency spectrum generated when an impulse reaches a boundary is independent of the distance from the generator. In this case the frequency is cutoff at roughly the size of the nerve impulse divided by the conduction velocity. Understanding this frequency behavior is important in that it forms the scientific basis for choosing filters to optimally record potentials with different origins. For instance, if recording of traveling potentials far from the axon, is desired, it will be important to keep low frequency (high pass) filters as low as possible. However, if the goal of an experiment is to analyze the changes in the local environment that a nerve impulse encounters, it is best to set the low frequency filter high enough to reduce the amplitude of the traveling potentials [30].

Finally, in a discussion of the possibility that radiation fields are generated during the propagation of neural impulses or when an impulse encounters a localized change in conductivity, it was demonstrated that such fields are extremely tiny. The main reasons for this are the fact that the rate of change in charge distributions is slow and the radiated power increases as the fourth power of the frequency. The radiated power also depends on the fourth power of the size of the generator and neural structures are typically small.

Many of the previous studies into the extracellular fields generated by action potentials were heavily reliant on actual recordings and on finite element simulations of the extracellular fields. This resulted in the empirical classification shown in Table 1. Both of these techniques are valuable but they do not give a full sense of the types of field generated that analytic computations can. The arguments presented in this paper strongly suggest that a better classification for extracellular fields should be that described in Table 3. This classification is based primarily on whether the recorded potentials are dipolar, quadrupolar or related to finite volume effects and whether they are generated by a traveling wave of excitation or generated when the traveling wave reaches a point where there is either a change in the axon geometry, axon cable properties or the local extracellular environment. This approach avoids the ambiguity associated with the terms near-field and far-field potential which have in the last 30 years been used in many different contexts by different authors.

**Table 3:** Summary of Extracellular Field Types Far From An Axon

**Field Type**	**Causes**	**Far-Field Behavior**	**Angular**	**Generator Type**	**Comment**
Radiation	Change in Dipole Direction Moving Dipole Through Region of Altered Electrical Properties		cos(*θ*) -Free Particle. Complex Angular Dependence for Transition Radiation	Traveling	Clinically Insignificant
Dipole	Change in Axon Radius (Dipole Tangent To Axon)		cos(*θ*)	Stationary	Includes "Sealed End" Axons
	Axon curvature (Dipole Normal To Axon)		cos(*θ*)	Stationary	
	Localized Changes in Extracellular Electrical Properties		Cos(*θ*)	Stationary	Only When Cylindrical Symmetry is Broken
Quadrupolar	Impulse Travelling Along Uniform, Straight, Homogenous Axon in Isotropic Electrical Environment.		3 cos^2^(*θ*)-1	Travelling	
	Changes in Cable Properties.		3 cos^2^(*θ*)-1	Stationary	
	Localized Changes in Extracellular Electrical Properties		3 cos^2^(*θ*)-1	Stationary	For Instance the Plane Conducting Boundary
Boundary	Fields Generated Because of the Finite Volume In Which The Generator is Immersed	Broadly Distributed	Depends Critically on Boundary Shape and Size	Stationary or Travelling	Potentials Linear Far From Source For A Cylindrical Volume Conductor

## Authors' contributions

SR participated in coordination of the study, designed and oversaw the modeling, and helped to draft the manuscript. DP and MI carried out the simulations. MS conceived of the study, participated in its design and coordination, aided in the derivations, and helped to draft the manuscript. All authors read and approved the final manuscript.

**Figure 15:**
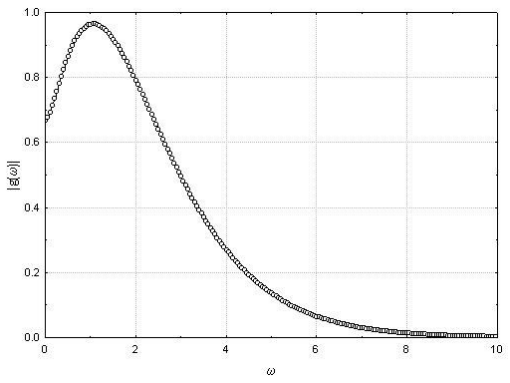
Plot of the function g(*ω*)in equation (E.14).

## Supplementary Material

Additional File 1 Appendices; with regards to p24, please see Figure 15 Click here for file
