# Supplementary material for: Classification of the extracellular fields produced by activated neural structures
Source: Biomed Eng Online. 2005 Sep 7;4:53. doi: 10.1186/1475-925X-4-53 (PMC1239920; doi:10.1186/1475-925X-4-53)
Supplement: Additional File 1 — Appendices; with regards to p24, please see Figure 15 [file 1475-925X-4-53-S1.doc]

**Appendices**

**Appendix A: Derivation of dipole moment and quadrupole moments associated with membrane depolarization.**

A surface in three dimensional space can be mathematically described as a vector function of two variables. In the following development, these two variables will be called$\left(s,\theta\right)$. It will be instructive to think of s as the distance along the length of the axon and $\theta$ as the angular position around the axon. It will be assumed that the membrane itself is very thin so that the axon can truly be represented by a surface rather than as a three dimensional structure. Prior to proceeding, some basic definitions will be useful:

$\vec{X}(s,\theta)$is the vector representing each point on the surface of the neural structure.

is the unit (outward) normal to the surface at $(s,\theta)$

The unit outward normal is determined from the position vector $\vec{X}(s,\theta)$ by the following construction [12]:

It is also well known that the unit of area dS on the surface is given by:

$$dS=\left|\frac{\partial\vec{X}(s,\theta)}{\partial\theta}\times\frac{\partial\vec{X}(s,\theta)}{\partial s}\right|d\theta ds$$

The transmembrane potential at a point $\left(s,\theta\right)$ on the surface of the axon will be called $V_{m}(s,\theta)$ and is related to the dipole moment density $\vec{d}_{m}(s,\theta)$ as follows [7]:

These considerations suggest the general expression for the extracellular field $V(\vec{x})$produced at an observation point$\vec{x}$:

$$V(\vec{x})=\frac{1}{4\pi}\left[\int_{0}^{L} \int_{0}^{2\pi} d\theta dsV_{m}(s,\theta)\left[\frac{\partial\vec{X}\left(s,\theta\right)}{\partial\theta}\times\frac{\partial\vec{X}\left(s,\theta\right)}{\partial s}\right]\bullet\left[\frac{\left(\vec{x}-\vec{X}(s,\theta)\right)}{\left|\vec{x}-\vec{X}(s,\theta)\right|^{3}}\right]\right]$$

where L is the length of the axon. In order to facilitate ensuing calculations, the following definitions will be used:

$$\begin{matrix} \vec{X}(s,\theta)=\vec{X}^{*}(s)+\vec{Y}(s,\theta) \\ \vec{X}^{*}(s)=\frac{1}{2\pi}\int_{0}^{2\pi} \vec{X}(s,\theta)d\theta\end{matrix}$$

where $\vec{X}^{*}(s)$is the curve describing the location of the centroid of the generalized axon as a function of s and $\vec{Y}(s,\theta)$is the vector pointing from the centroid at s to that part of the surface characterized by s and angular location $\theta$. At this point, it is helpful to divide the extracellular potentials into those that are dipolar and those that are quadrupolar at large distances from the neural structure. Assuming that the electrodes are many axon radii away from the axon:

$$\left[\frac{\left|\vec{Y}(s,\theta)\right|}{\left|\vec{x}\right|}\right]<<1$$

it is reasonable to simplify (A.4) through a Taylor series expansion:

$$\left[\frac{\left(\vec{x}-\vec{X}(s,\theta)\right)}{\left|\vec{x}-\vec{X}(s,\theta)\right|^{3}}\right]\approx\left[\frac{\left(\vec{x}-\vec{X}^{*}(s)\right)}{\left|\vec{x}-\vec{X}^{*}(s)\right|^{3}}\right]-\left(\vec{Y}(s,\theta)\bullet\vec{\nabla}_{\vec{x}}\right)\left[\frac{\left(\vec{x}-\vec{X}^{*}(s)\right)}{\left|\vec{x}-\vec{X}^{*}(s)\right|^{3}}\right]+...$$

to obtain a separation of the total extracellular potential at$\vec{x}$into dipolar, $V^{d}(\vec{x})$, and quadrupolar, $V^{q}(\vec{x})$, components:

$$V(\vec{x})=V^{d}(\vec{x})+V^{q}(\vec{x})$$

where:

$$V^{d}(\vec{x})=\frac{1}{4\pi}\left[\int_{0}^{L} \int_{0}^{2\pi} d\theta dsV_{m}(s,\theta)\left[\frac{\partial\vec{X}\left(s,\theta\right)}{\partial\theta}\times\frac{\partial\vec{X}\left(s,\theta\right)}{\partial s}\right]\bullet\left[\frac{\left(\vec{x}-\vec{X}^{*}(s)\right)}{\left|\vec{x}-\vec{X}^{*}(s)\right|^{3}}\right]\right]$$

and:

$$V^{q}(\vec{x})=\frac{1}{4\pi}\left[\int_{0}^{L} \int_{0}^{2\pi} d\theta dsV_{m}(s,\theta)\left[\frac{\partial\vec{X}\left(s,\theta\right)}{\partial\theta}\times\frac{\partial\vec{X}\left(s,\theta\right)}{\partial s}\right]\bullet\left\{\vec{Y}(s,\theta)\bullet\vec{\nabla}_{x}\left[\frac{\left(\vec{x}-\vec{X}^{*}(s)\right)}{\left|\vec{x}-\vec{X}^{*}(s)\right|^{3}}\right]\right\}\right]$$

Contributions from higher order moments can be derived by carrying out the Taylor series expansion to higher orders but these are less important in clinical applications. In order to understand the fields generated by impulses propagating down the nerve, it is important to estimate the contribution made by each small membrane element activated along the course of the neural structure. According to (A.8), a membrane segment at s has an effective dipole moment per unit change in s of

$$\vec{d}(s)=\frac{1}{4\pi}\int_{0}^{2\pi} \left[\frac{\partial\vec{X}(s,\theta)}{\partial\theta}\times\frac{\partial\vec{X}(s,\theta)}{\partial s}\right]V_{m}(s,\theta)d\theta$$

so that

$$V^{d}(\vec{x})=\int_{0}^{L} ds\vec{d}(s)\bullet\left[\frac{\left(\vec{x}-\vec{X}^{*}(s)\right)}{\left|\vec{x}-\vec{X}^{*}(s)\right|^{3}}\right]$$

and a quadrupole moment per unit length of activated axon:

$$\overleftrightarrow{Q}(s)=-\frac{1}{4\pi}\int_{0}^{L} \int_{0}^{2\pi} \left[\frac{\partial\vec{X}(s,\theta)}{\partial\theta}\times\frac{\partial\vec{X}(s,\theta)}{\partial s}\right]\vec{Y}(s,\theta)V_{m}(s,\theta)d\theta ds$$

with the corresponding potential:

$$V_{tot}^{q}(s)=\int_{0}^{L} \overleftrightarrow{Q}(s)\bullet\vec{\nabla}_{\vec{x}}\left[\frac{\left(\vec{x}-\vec{X}^{*}(s)\right)}{\left|\vec{x}-\vec{X}^{*}(s)\right|^{3}}\right]ds$$

Consider a generalized neural structure that is locally a thin tube surrounding its centroid with an angle dependent radius$a(s,\theta)$. In this case, it is possible to write:

where are the unit tangent, normal and binormal vectors to the curve $\vec{X}^{*}(s)$at s and s is taken as the arc-length along the curve $\vec{X}^{*}(s)$. $\kappa^{*}(s)$is the curvature [12] of $\vec{X}^{*}(s)$at s. This enables a computation of the derivatives required to compute the dipole and quadrupole moments:

This can be simplified using the Serret-Frenet formulae [12]:

where $\tau^{*}(s)$is the torsion [12] of $\vec{X}^{*}(s)$at s, to yield:

Similarly, it is straightforward to demonstrate:

Using the following expressions:

it is possible to deduce that:

Using integration by parts, it can be shown that:

where:

$$a_{0}^{2}(s)=\frac{1}{2\pi}\int_{0}^{2\pi} a(s,\theta)^{2}d\theta$$

Thus, if the transmembrane potential$V_{m}\left(s,\theta\right)=V_{m}(s)$ is independent of $\theta$:

and:

$$\left|\vec{d}(s)\right|=\frac{1}{4}V_{m}(s)\sqrt{\left[\frac{\partial a_{0}^{2}(s)}{\partial s}\right]^{2}+\left[a_{0}^{2}(s)\kappa^{*}(s)\right]^{2}}$$

The first step in evaluating the quadrupole moment involves writing (A.12) and (A.13) in their coordinate forms:

$$Q_{ij}(s)=-\frac{1}{4\pi}\int_{0}^{2\pi} \left[\frac{\partial\vec{X}(s,\theta)}{\partial\theta}\times\frac{\partial\vec{X}(s,\theta)}{\partial s}\right]_{i}\vec{Y}(s,\theta)_{j}V_{m}(s,\theta)d\theta$$

so that:

$$V^{q}(\vec{x})=-\int_{0}^{L} Q_{ij}(s)\frac{\partial^{2}}{\partial x_{i}\partial x_{j}}\left[\frac{1}{\left|\vec{x}-\vec{X}^{*}(s)\right|}\right]ds$$

It is possible to write:

Since:

$$\frac{\partial^{2}}{\partial x_{i}\partial x_{i}}\left[\frac{1}{\left|\vec{x}-\vec{X}^{*}(s)\right|}\right]=\frac{\partial^{2}}{\partial x_{j}\partial x_{i}}\left[\frac{1}{\left|\vec{x}-\vec{X}^{*}(s)\right|}\right]$$

only the symmetric component:

$$Q_{ij}^{s}(s)=\frac{1}{2}\left(Q_{ij}(s)+Q_{ji}(s)\right)$$

of the quadrupole tensor contributes to the extracellular field. Carrying out the calculations below with a heavy use of integration by parts, the symmetric quadrupole moment tensor can be computed as: $Q_{ij}^{S}(s)=-\frac{1}{2}a_{0}^{2}(s)V_{m}(s)\left[\begin{matrix} 0 & -\frac{1}{3a_{0}^{2}(s)}\left[\frac{d\left(a_{c}^{3}(s)\right)}{ds}-\tau^{*}(s)a_{s}^{3}(s)\right] & -\frac{1}{3a_{0}^{2}(s)}\left[\frac{da_{s}^{3}}{ds}+\tau^{*}(s)a_{c}^{3}(s)\right] \\ -\frac{1}{3a_{0}^{2}(s)}\left[\frac{d\left(a_{c}^{3}(s)\right)}{ds}-\tau^{*}(s)a_{s}^{3}(s)\right] & 1-\frac{4}{3}\kappa^{*}(s)\frac{a_{c}^{3}(s)}{a_{0}^{2}(s)} & \frac{1}{3}\kappa^{*}(s)\frac{a_{s}^{3}(s)}{a_{0}^{2}(s)} \\ -\frac{1}{3a_{0}^{2}(s)}\left[\frac{d\left(a_{s}^{3}(s)\right)}{ds}+\tau^{*}(s)a_{c}^{3}(s)\right] & \frac{1}{3}\kappa^{*}(s)\frac{a_{s}^{3}(s)}{a_{0}^{2}(s)} & 1-\frac{2}{3}\kappa^{*}(s)\frac{a_{c}^{3}(s)}{a_{0}^{2}(s)} \end{matrix}\right]$

in the Frenet frame consisting of the three basis vectors which correspond to index values of 1, 2, and 3 respectively. Tensor transformation formulae can then be used to compute Q^S^ in any other coordinate system. The following definitions have been used to simplify the notation:

$$\begin{matrix} a_{c}^{3}(s)=\frac{1}{2\pi}\int_{0}^{2\pi} a(s,\theta)^{3}\left(\cos(\theta)\right)d\theta\\ a_{s}^{3}(s)=\frac{1}{2\pi}\int_{0}^{2\pi} a(s,\theta)^{3}\left(\sin(\theta)\right)d\theta\end{matrix}$$

In order to understand the above expression in more detail, it is helpful to note that, in the case where the axon cross section has inversion symmetry around its centroid, the expression for the quadrupole moment simplifies to:

$$Q^{s}(s)=-\frac{1}{2}a_{0}^{2}(s)V_{m}(s)\left[\begin{matrix} 0 & 0 & 0 \\ 0 & 1 & 0 \\ 0 & 0 & 1 \end{matrix}\right]$$

The leading-trailing dipole model of extracellular fields [13], however, suggests that Q^S^ should have components only along the long axis of the axon. The result (A.32) does not contradict the leading-trailing dipole model. This can be easily seen since the Laplace equation states:

and so it is possible to subtract any diagonal tensor from the above expression for the quadrupole moment tensor without changing the computed fields. Thus, the extracellular fields produced by a quadrupole moment $Q^{s}(s)$are the same as those generated by a quadrupole moment of the form:

$$Q_{ij}^{S}(s)=-\frac{1}{2}a_{0}^{2}V_{m}(s)\left[\begin{matrix} -1 & 0 & 0 \\ 0 & 0 & 0 \\ 0 & 0 & 0 \end{matrix}\right]$$

which is what is expected on the basis of the leading-trailing dipole model. As a simple application, it is useful to determine how the extracellular fields are changed when an axon is uniformly distorted along its length:

$$a(s,\theta)=r_{0}+r_{1}\cos^{2}(\theta)$$

This implies:

$$a_{0}^{2}=r_{0}^{2}+r_{0}r_{1}+\frac{3}{8}r_{1}^{2}$$

so that:

$\overleftrightarrow{Q}(s)=-\frac{1}{2}\left(r_{0}^{2}+r_{0}r_{1}+\frac{3}{8}r_{1}^{2}\right)V_{m}(s)\left[\begin{matrix} 0 & 0 & 0 \\ 0 & 1 & 0 \\ 0 & 0 & 1 \end{matrix}\right]$ (A.36)

If the axon is nearly completely flattened, $r_{1}=-r_{0}$ and the magnitude of the quadrupole moment is reduced by a factor of $\frac{3}{8}$from the baseline value. Thus, flattening a localized region of an axon is associated with a local decrease in the amplitude of the extracellular fields as this region is traversed by an action potential. However, neither the directionality or the quadrupolar character of the field is changed. Of course, a dipolar field is generated as the action potential enters and exits the region of altered axonal diameter according to equation (A.23).

**Appendix B: Derivation of fields generated by small conducting sphere in arbitrary electric field**

Assume that the electric potential over all space prior to the introduction of the conducting sphere is given by $\phi_{0}(\vec{x})$. An uncharged, conducting sphere of radius a is then placed in this field at position $\vec{x}=0$, and it is desired to compute the resultant electric field. Now the field generated by the conducting sphere $\phi_{s}(r,\theta,\varphi)$(r is the distance from the center of the sphere, $\theta$ is the polar angle and $\varphi$is the azimuthal angle) must be of the form:

$$\phi_{s}(r,\theta,\varphi)=\sum_{l=1}^{\infty} \sum_{m=-l}^{l} A_{lm}r^{-(l+1)}Y_{lm}(\theta,\varphi)$$

where the $Y_{lm}(\theta,\varphi)$are the spherical harmonics. Note that the smallest value of l must be 1 since the sphere is uncharged. Because the sphere is conducting, its surface must be an isopotential and so:

$$\phi_{s}(a,\theta,\varphi)+\phi_{0}(a,\theta,\varphi)=V_{s}$$

Where V_s_ is the potential of the sphere. Using the orthogonality of the spherical harmonics:

$$\begin{matrix} \int d\Omega Y_{l'm'}(\theta,\varphi)Y_{lm}^{*}(\theta,\varphi)=\delta_{ll'}\delta_{mm'} \\ d\Omega=\sin(\theta)d\theta d\varphi\end{matrix}$$

It is possible to write:

$$\frac{\int d\Omega\phi_{0}(a,\theta,\varphi)Y_{00}^{*}(\theta,\varphi)}{\int d\Omega Y_{00}^{*}(\theta,\varphi)}=V_{s}$$

and:

$$a^{l+1}\int d\Omega\phi_{0}(a,\theta,\varphi)Y_{lm}^{*}(\theta,\varphi)=-A_{lm}$$

where the integrals are over the surface of the sphere r=a. These equations have some interesting consequences. Note, in particular, that there is a dipolar field (l=1) generated by the sphere as long as:

$$\int d\Omega\phi_{0}(a,\theta,\varphi)Y_{1m}^{*}(\theta,\varphi)\neq0$$

In order to estimate the actual induced dipole moment (B.5) can be rewritten as:

$$a^{2}\int_{0}^{\pi} \int_{0}^{2\pi} \phi_{0}(a,\theta,\varphi)Y_{1m}^{*}(\theta,\varphi)\sin\left(\theta\right)d\theta d\varphi=-A_{1m}$$

where the integral is over the surface of the conducting sphere so that:

where$\vec{x}_{0}$is the coordinate of the center of the sphere. If the sphere is small, a Taylor series expansion can be used to obtain:

where:

Thus, an approximate expression for A_1m_ of the sphere is:

$$a^{2}\int_{0}^{\rho} \int_{0}^{2\pi} \vec{\varepsilon}\bullet\vec{\nabla}\varphi_{0}\left|_{\vec{x}=\vec{x}_{0}}Y_{1m}^{*}\left(\theta,\varphi\right)\sin\left(\theta\right)d\theta d\varphi=-A_{1m}\right.$$

This implies that:

$$\begin{matrix} A_{1m}^{1}=-a^{3}\left.\frac{\partial\phi_{0}}{\partial z}\right|_{\vec{x}=\vec{x}_{0}}\int_{0}^{\pi} \int_{0}^{2\pi} \cos(\theta)Y_{1m}^{*}(\theta,\varphi)\sin(\theta)d\theta d\varphi\\ A_{1m}^{2}=-a^{3}\left.\frac{\partial\phi_{0}}{\partial x}\right|_{\vec{x}=\vec{x}_{0}}\int_{0}^{\pi} \int_{0}^{2\pi} \sin(\theta)\cos(\varphi)Y_{1m}^{*}(\theta,\varphi)\sin(\theta)d\theta d\varphi\\ A_{1m}^{3}=-a^{3}\left.\frac{\partial\phi_{0}}{\partial y}\right|_{\vec{x}=\vec{x}_{0}}\int_{0}^{\pi} \int_{0}^{2\pi} \sin(\theta)\sin(\varphi)Y_{1m}^{*}(\theta,\varphi)\sin(\theta)d\theta d\varphi\\ A_{1m}=A_{1m}^{1}+A_{1m}^{2}+A_{1m}^{3} \end{matrix}$$

Substituting the values of the spherical harmonics it can be shown that the dipole contribution to the total potential induced by the presence of the sphere is: $\begin{matrix} \phi_{s}^{d}(r,\theta,\varphi)=\frac{\sum_{m=-1}^{1} \left(A_{1m}^{1}+A_{1m}^{2}+A_{1m}^{3}\right)Y_{1m}(\theta,\varphi)}{r^{2}} \\ =-\frac{a^{3}\left[\left.\frac{\partial\phi_{0}}{\partial z}\right|_{\vec{x}=\vec{x}_{0}}\cos\left(\theta\right)\left.+\frac{\partial\phi_{0}}{\partial x}\right|_{\vec{x}=\vec{x}_{0}}\sin\left(\theta\right)\cos\left(\varphi\right)\left.+\frac{\partial\phi_{0}}{\partial y}\right|_{\vec{x}=\vec{x}_{0}}\sin\left(\theta\right)\sin\left(\varphi\right)\right]}{r^{2}} \end{matrix}$

This implies that even if the external potential is generated by a quadrupolar source, the presence of a small conducting sphere will produce a dipolar field when the electric field is non-zero at the location of the sphere. This result was also derived by Jewett [35] in the case of extracellular recording and by Roth [14] in the case of nerve stimulation. Noting that:

$$\vec{E}_{0}=-\vec{\nabla}\phi_{0}\left(\vec{x}\right)$$

it becomes possible to write:

(B.17)

This argument can be extended to other situations. In particular, it is well known [16] that the dipole moment induced in any dielectric object in an external electric field is:

(B.18)

where v is the volume of the object and $\overleftrightarrow{\zeta}$is a constant tensor that depends on the geometry of the object and the difference between the dielectric constant (or conductivity) of the object and the medium. For a dielectric sphere whose size is much smaller than the distance over which the imposed external field changes significantly:

$\zeta_{ij}=3\frac{\frac{\varepsilon}{\varepsilon_{0}}-1}{\frac{\varepsilon}{\varepsilon_{0}}+2}\delta_{ij}$ (B.19)

where $\varepsilon$ is the dielectric constant of the sphere,$\varepsilon_{0}$is the dielectric constant of the rest of the medium and $\delta_{ij}$is the Kronecker delta. For a sphere with conductivity differing from that of the remainder of the environment, it is also true that:

$\zeta_{ij}=3\frac{\frac{\sigma}{\sigma_{0}}-1}{\frac{\sigma}{\sigma_{0}}+2}\delta_{ij}$ (B.20)

where$\sigma$ is the conductivity of the sphere and$\sigma_{0}$is the conductivity of the rest of the medium.

**Appendix C: Effects finite a volume conductor on the recorded extracellular fields**

The following discussion will be based in cylindrical coordinates and will refer to a cylinder of radius a whose long axis is taken as the z axis. r will be the radial distance from the axis, $\theta$ will be the polar angle. It is assumed that all charges are confined to a finite segment of the cylinder $z_{0}-\Delta<z<z_{0}+\Delta$. The Poisson equation for this system is:

$$\begin{matrix} \nabla^{2}\varphi\left(r,\theta,z\right)=-4\pi\rho\left(r,\theta,z\right) \\ \rho\left(r,\theta,z\right)=0;z<z_{0}-\Delta\\ \rho\left(r,\theta,z\right)=0;z>z_{0}+\Delta\end{matrix}$$

Analysis of this problem begins by considering the potentials in the regions that are free of charge:

$$\begin{matrix} \varphi_{<}\left(r,\theta,z\right)=\varphi\left(r,\theta,z\right);z<z_{0}-\Delta\\ \varphi_{>}\left(r,\theta,z\right)=\varphi\left(r,\theta,z\right);z>z_{0}+\Delta\end{matrix}$$

so that:

$$\begin{matrix} \nabla^{2}\varphi_{<}(r,\theta,z)=0;z>z_{0}-\Delta\\ \nabla^{2}\varphi_{>}(r,\theta,z)=0;z>z_{0}+\Delta\end{matrix}$$

Assume that on the surface $r=a$, the following boundary condition applies

$$\left.\frac{\partial\varphi}{\partial r}\right|_{r=a}=-k\varphi$$

This would be the case if there were a thin resistive coating on the surface of the cylinder with k inversely proportional to that resistance. When k=0, the boundary is totally insulating. At a later point, the effects of boundary conditions at the ends of the cylinder will be explored. Note that in cylindrical coordinates:

$$\nabla^{2}\varphi=\frac{1}{r}\frac{\partial}{\partial r}\left(r\frac{\partial\varphi}{\partial r}\right)+\frac{1}{r^{2}}\frac{\partial^{2}\varphi}{\partial\theta^{2}}+\frac{\partial^{2}\varphi}{\partial z^{2}}$$

In the region where there are no charges, the integrals:

$$\begin{matrix} \tilde{\varphi}_{>}(z)=\frac{\int_{0}^{2\pi} \int_{0}^{a} 2\pi r\varphi_{>}\left(r,\theta,z\right)drd\theta}{\int_{0}^{2\pi} \int_{0}^{a} rdrd\theta} \\ \tilde{\varphi}_{<}(z)=\frac{\int_{0}^{2\pi} \int_{0}^{a} 2\pi r\varphi_{<}\left(r,\theta,z\right)drd\theta}{\int_{0}^{2\pi} \int_{0}^{a} rdrd\theta} \end{matrix}$$

which describe the average of the potential at a given axial location z will be well defined. Integrating the Poisson equation, it becomes possible to write:

$$\begin{matrix} \int_{0}^{2\pi} \frac{1}{r^{2}}\frac{\partial^{2}\varphi}{\partial\theta^{2}}d\theta=0 \\ \int_{0}^{a} 2\pi r\left[\frac{1}{r}\frac{\partial}{\partial r}\left(r\frac{\partial\varphi}{\partial r}\right)\right]dr=\left.\left(r\frac{\partial\varphi}{\partial r}\right)\right|_{r=a}-\mathop{Lim}\limits_{\xi\to0} \left.\left(r\frac{\partial\varphi}{\partial r}\right)\right|_{r=\xi} \end{matrix}$$

Now, as long as only regions where there is no charge are being considered:

$$\mathop{Lim}\limits_{\xi\to0} \left.\left(r\frac{\partial\varphi}{\partial r}\right)\right|_{r=\xi}=0$$

so that using the boundary condition at the cylinder surface:

$$\int_{0}^{a} 2\pi r\left[\frac{1}{r}\frac{\partial}{\partial r}\left(r\frac{\partial\varphi}{\partial r}\right)\right]dr=ka\varphi(a,\theta,z)$$

In the case where k=0 it is then possible to write the expressions for the averaged potential in the source free region as:

$$\begin{matrix} \frac{d^{2}\tilde{\varphi}_{<}(z)}{dz^{2}}=0;z<z_{0}-\Delta\\ \frac{d^{2}\tilde{\varphi}_{>}(z)}{dz^{2}}=0;z>z_{0}+\Delta\end{matrix}$$

so that the averaged potentials on either side of the charged region must be of the form:

$$\begin{matrix} \tilde{\varphi}_{>}=a_{>}+b_{>}z \\ \tilde{\varphi}_{<}=a_{<}+b_{<}z \end{matrix}$$

It is possible to apply Gauss’s law to a surface enclosing the region in which the charges reside. Contributions from the cylindrical surface vanish since those boundaries were taken as insulating. The remainder of the contribution is from the two ends:

$\left.\left.\frac{d\tilde{\varphi}_{>}(z)}{dz}\right|_{z>z_{0}}-\frac{d\tilde{\varphi}_{<}(z)}{dz}\right|_{z<z_{0}}=4\pi Q$ (C.15)

thus:

$b_{>}=b_{<}+4\pi Q$ (C.16)

Note that since both slopes cannot be zero in the presence of net charge, it is impossible to impose insulating conditions at both ends of the cylinder. This issue with Neumann conditions is well known and discussed in Morse and Feshbach [31]. Note this conclusion that the potentials are linear functions of z outside the region of charges is not dependent on their actual distribution as long as they are distributed only over a finite region of the cylinder. What type of potentials do we expect if the source is dipolar or quadrupolar? Clearly, in these cases $b_{<}=b_{>}$and if insulating conditions are placed at the ends of the cylinder both must be zero. Thus the averaged potential must be constant outside the area where the charges are located for either a dipolar or quadrupolar source although that constant may be different in these two cases. In order to estimate the difference in these constant potentials, it is instructive to look at the following integral of the exact Poisson equation:

$\int_{0}^{2\pi} d\theta\int_{0}^{a} rdr\int_{z_{0}-\Delta}^{z_{0}+\Delta} (z-z_{0})dz\nabla^{2}\varphi\left(r,\theta,z\right)=-4\pi d_{z}$ (C.17)

where d_z_ is the z component of the dipole moment of the charge density. Simplifying, integrating by parts and noting that as long as the net charge is zero the z derivative of the averaged potential is zero at the edges of the boundary it is possible to write:

$\int_{z_{0}-\Delta}^{z_{0}+\Delta} (z-z_{0})dz\frac{d^{2}\tilde{\varphi}\left(z\right)}{dz^{2}}=-\left[\tilde{\varphi}(z_{0}+\Delta)-\tilde{\varphi}(z_{0}-\Delta)\right]=a_{<}-a_{>}$ (C.18)

Thus:

$a_{>}=a_{<}+4\pi d_{z}$ (C.19)

and so the difference in potential is proportional to the component of the dipole moment along the cylinder axis. Following the above argument, purely quadrupolar sources would not be expected to generate extracellular potentials far from the source within a cylindrical volume conductor of uniform conductivity. These results are similar to those obtainable using the Green’s function for the appropriate cylindrical boundary conditions [17].

Within the above approach it is possible to qualitatively estimate how deviations from perfect insulation affect the potentials far from the source**.** Returning to the derivation adduced above, it is seen that in the case $k\neq0$ the integrated Poisson equation becomes**:**

$\begin{matrix} \frac{d^{2}\tilde{\varphi}_{<}(z)}{dz^{2}}=\frac{k}{\pi a}\varphi_{<}(a,z);z<z_{0}-\Delta\\ \frac{d^{2}\tilde{\varphi}_{>}(z)}{dz^{2}}=\frac{k}{\pi a}\varphi_{>}(a,z);z>z_{0}+\Delta\end{matrix}$ (C.20)

where $\varphi(a,z)$ is the averaged potential on the surface of the cylinder as a function of z:

$\varphi(a,z)=\frac{\int_{0}^{2\pi} \varphi(a,\theta,z)d\theta}{2\pi}$ (C.21)

It is not unreasonable to assume as a first approximation for a thin cylinder that this is proportional to:

$\frac{\int_{0}^{2\pi} \int_{0}^{a} r\varphi\left(r,\theta,z\right)drd\theta}{\int_{0}^{2\pi} \int_{0}^{a} rdrd\theta}$ (C.22)

the averaged potential over the entire cross section:

$\frac{\int_{0}^{2\pi} \int_{0}^{a} r\varphi_{<}\left(r,\theta,z\right)drd\theta}{\int_{0}^{2\pi} \int_{0}^{a} rdrd\theta}\approx\alpha\frac{\int_{0}^{2\pi} \varphi(a,\theta,z)d\theta}{2\pi}$ (C.23)

for some constant $\alpha$. This implies that

$\varphi(a,z)=\alpha\tilde{\varphi}(z)$ (C.24)

$\begin{matrix} \frac{d^{2}\tilde{\varphi}_{<}(z)}{dz^{2}}=\frac{k}{\pi a}\alpha\tilde{\varphi}_{>}(z);z<z_{0}-\Delta\\ \frac{d^{2}\tilde{\varphi}_{>}(z)}{dz^{2}}=\frac{k}{\pi a}\alpha\tilde{\varphi}_{<}(z);z>z_{0}+\Delta\end{matrix}$ (C.25)

The solutions to this problem that are not divergent are:

$\begin{matrix} \tilde{\varphi}_{<}(z)=c_{<}e^{z\sqrt{\frac{k\alpha}{\pi a}}};z<z_{0}-\Delta\\ \tilde{\varphi}_{<}(z)=d_{<}e^{-z\sqrt{\frac{k\alpha}{\pi a}}};z>z_{0}+\Delta\end{matrix}$ (C.26)

Integrating over the region in which the charges are contained and imposing boundary conditions at the ends of the cylinder can determine the relative value of the above coefficients. The key observation is that the character of the potentials are changed entirely removing the strict insulating conditions on the surface of the cylinder. The distance over which the potentials change is on the order of$\sqrt{\frac{\pi a}{k\alpha}}$and so the recorded potentials are EXTREMELY dependent on the exact nature of the boundary conditions especially when k is small.

In addition, it should be noted that on its face, this argument does not explain the transition to the case of an infinite volume conductor as its radius becomes large. A simple variational argument provides some insight into this issue. Consider a potential of the form

$\phi(r,z)=A(r)B(z)$ (C.27)

Assuming for the moment that a reasonable choice for A(r) can be chosen a priori, what is the optimal choice of B(z)? In the charge free region, the variational principle states that it is the function which is associated with the minimum energy:

$E\approx\int_{0}^{a} rdr\int_{\Delta}^{\infty} dz\left|\vec{\nabla}\phi\right|^{2}=\int_{0}^{a} rdrA'(r)^{2}\int_{\Delta}^{\infty} dzB(z)^{2}+\int_{0}^{a} rdrA(r)^{2}\int_{\Delta}^{\infty} dzB'(z)^{2}$ (C.28)

This is a simple variational problem with the solution:

$\begin{matrix} B''(z)=B(z)\varsigma\\ \varsigma=\frac{\int_{0}^{a} rdrA'(r)^{2}}{\int_{0}^{a} rdrA(r)^{2}} \end{matrix}$ (C.29)

This implies that when the distribution of potential is relatively constant across a cross section of the cylinder as might be expected many cylinder diameters from the charges $\varsigma\to0$and so the z dependence of the potential is linear. If A(r) changes over a characteristic distance $\tilde{a}$ then $\varsigma\sim\frac{1}{\tilde{a}^{2}}$and the potential in the z direction decays as $e^{-\frac{z}{\tilde{a}}}$. Of course, very close to the charges, the expression for $\varsigma$is divergent and so this argument does not apply.

The picture is then that for r<<a, z<<a, the potential generated by charges in the finite volume conductor is similar to that in an infinite volume conductor. When z>>a the potentials change in a linear fashion with z and have a roughly constant radial profile.

**Appendix D: Illustration of the effects of changing axon cable properties on recorded extracellular fields**

In order to illustrate the effects that a sudden change in the cable properties of an axon might have on the recorded extracellular fields, a number of simple simulations were undertaken. In each case the response of a straight cable-like axon to a point stimulator moving with constant velocity along the axon membrane is studied. This is a simple model used to study extracellular fields and is not a realistic model of the propagation of an action potential. Define$r_{m}(z)$as the membrane resistance, $r_{i}(z)$as the internal resistance, and $c_{m}(z)$ as the membrane capacitance at each point along the axon. Then, in the case of a stimulator which injects a constant transmembrane current I_o_ at the point of contact, the generalized cable equation determining the transmembrane potential $V_{m}$ becomes:

$r_{m}(z)\frac{\partial}{\partial z}\left[\frac{1}{r_{i}\left(z\right)}\frac{\partial V_{m}}{\partial z}\right]-\tau\left(z\right)\frac{\partial V_{m}}{\partial t}-V_{m}=r_{m}(z)I_{0}\delta\left(z-\text{v}t\right)$ (D.1)

where:

$$\lambda\left(z\right)\approx\sqrt{\frac{r_{m}\left(z\right)}{r_{i}\left(z\right)}}$$

is the space constant at each point along the axon and:

$$\tau\left(z\right)=r_{m}\left(z\right)c_{m}\left(z\right)$$

is the local time constant [2]. Solutions to these equations were obtained using finite element modeling in Femlab (Comsol, Natick MA). All solutions were carried out in a linear domain extending from z=-100 mm to z=250 mm in 960 spatial grid elements. The impulse was presumed to move at a velocity of 40 m/sec. At time t=0 the stimulator was at the point z=0. The simulation began at time t=-2.5 msec and continued in steps of .01msec to 5.0 msec. Data was then exported into a Fortran program (Absoft, Rochester Hills, MI) that was used to compute the extracellular potentials at given locations as a function of time. From the transmembrane potentials, the extracellular potentials were computed in the following way. The axon was divided into small segments equal to the grid size from the finite element simulation. The potential from each small segment was taken as that of the quadrupole associated with the mean transmembrane potential in each small segment. The total potential was taken as the superposition of the fields generated by each small segment. It should be noted that dipolar potentials cannot be generated in this model no matter what the form of the above equation as long as the cable-like axon is cylindrical.

Simulations were carried out under the condition that the membrane resistance suddenly quintuples at the point z=100:

$\begin{matrix} \tau\left(z\right)=\left(0.5\text{msec}\right)\left[1+2\left(1+\tanh\left(\frac{z-100}{.1}\right)\right)\right] \\ \lambda^{2}\left(z\right)=\left\{\left(2.5\text{mm}\right)^{2}\left[1+2\left(1+\tanh\left(\frac{z-100}{.1}\right)\right)\right]\right\} \\ r_{m}\left(z\right)=r_{m}\left(-\infty\right)\left[1+2\left(1+\tanh\left(\frac{z-100}{.1}\right)\right)\right] \end{matrix}$ (D.2)

The results of this simulation are demonstrated in Figure 11. It should be noted that a biphasic not triphasic extracellular potential is seen in this simulation when the recording electrode is close to the axon. This is due to the very long length of the depolarized segment $\left(\text{v}*\tau\left(z\right)\right)$.

**Appendix E: Frequency spectrum of recorded action potentials**

Equation (A.13) provides the relationship between the recorded extracellular potential and the quadrupole moment of the impulse:

$V_{tot}^{q}(s)=\int_{0}^{L} Q(s)\bullet\vec{\nabla}_{\vec{x}}\left[\frac{\left(\vec{x}-\vec{X}^{*}(s)\right)}{\left|\vec{x}-\vec{X}^{*}(s)\right|^{3}}\right]ds$ (E.1)

If the axon is linear and of infinite length, it is possible to write:

$V_{tot}^{q}(\vec{x},t)=\int_{-\infty}^{\infty} Q(s,t)\bullet\vec{\nabla}_{\vec{x}}\left[\frac{\left(\vec{x}-\vec{X}^{*}(s)\right)}{\left|\vec{x}-\vec{X}^{*}(s)\right|^{3}}\right]ds$ (E.2)

In order to further understand the spectra recorded from a traveling action potential, it is appropriate to consider the case in which:

$Q(s,t)=\alpha(s)q\left(\frac{s-\text{v}t}{\beta}\right)$ (E.3)

where v is the velocity of the nerve impulse, q(t) is a scalar function describing the distribution of the quadrupole moment around its peak values (ie the shape of the action potential) with width $\beta$and$\alpha(s)$is a constant tensor. Constructing the Fourier transform:

$\begin{matrix} V_{tot}^{q}(\vec{x},\omega)=\frac{1}{\sqrt{2\pi}}\int_{-\infty}^{\infty} e^{-i\omega t}V_{tot}^{q}(\vec{x},t)dt \\ V_{tot}^{q}(\vec{x},t)=\frac{1}{\sqrt{2\pi}}\int_{-\infty}^{\infty} e^{i\omega t}V_{tot}^{q}(\vec{x},\omega)d\omega\end{matrix}$ (E.4)

and making use of the change of variables:

$$y=\frac{s-\text{v}t}{\beta}$$

it is possible to write:

$Q(s,\omega)=\frac{\beta\alpha(s)}{\text{v}}e^{-i\omega\frac{s}{\text{v}}}\tilde{q}\left(\frac{\beta\omega}{\text{v}}\right)$ (E.5)

where:

$\tilde{q}(\omega)=\frac{1}{\sqrt{2\pi}}\int_{-\infty}^{\infty} q(t)e^{-i\omega t}dt$ (E.6)

$V_{tot}^{q}(\vec{x},\omega)=\int_{-\infty}^{\infty} Q(s,\omega)\bullet\vec{\nabla}_{\vec{x}}\left[\frac{\left(\vec{x}-\vec{X}^{*}(s)\right)}{\left|\vec{x}-\vec{X}^{*}(s)\right|^{3}}\right]ds$ (E.7)

According to previous discussions regarding the quadrupole moment tensor, $\alpha(s)$can be chosen to be zero except for the zz component so that:

$V_{tot}^{q}(\vec{x},\omega)=-\int_{-\infty}^{\infty} \frac{\beta\alpha(s)}{\text{v}}e^{-i\omega\frac{s}{\text{v}}}\tilde{q}\left(\frac{\beta\omega}{\text{v}}\right)\frac{\partial}{\partial z}\frac{\partial}{\partial z}\left[\frac{1}{\left|\vec{x}-\vec{X}^{*}(s)\right|}\right]ds$ (E.8)

moreover since:

it can be shown that:

$V_{tot}^{q}(\vec{x},\omega)=\tilde{q}\left(\frac{\beta\omega}{\text{v}}\right)\int_{-\infty}^{\infty} \frac{\beta\alpha(s)}{\text{v}}e^{-i\omega\frac{s}{\text{v}}}\left\{\frac{3(z-s)^{2}}{\left(x^{2}+y^{2}+(z-s)^{2}\right)^{\frac{5}{2}}}-\frac{1}{\left(x^{2}+y^{2}+(z-s)^{2}\right)^{\frac{3}{2}}}\right\}ds$ (E.9)

Defining:

$$R^{2}=x^{2}+y^{2}$$

it is possible to show that:

$V_{tot}^{q}(\vec{x},\omega)=\frac{1}{R^{3}}\tilde{q}\left(\frac{\beta\omega}{\text{v}}\right)\frac{\beta\alpha}{\text{v}}\int_{-\infty}^{\infty} e^{-i\omega\frac{s}{\text{v}}}\left\{\frac{2\frac{(z-s)^{2}}{R^{2}}-1}{\left(1+\frac{(z-s)}{R^{2}}^{2}\right)^{\frac{5}{2}}}\right\}ds$ (E.10)

For the sake of illustration, assume that the distribution of transmembrane potentials was Gaussian, in this case:

$\begin{matrix} q(x)=\frac{1}{\sqrt{2\pi}}e^{-\frac{1}{2}x^{2}} \\ \tilde{q}(x)=\frac{1}{\sqrt{2\pi}}e^{-\frac{1}{2}x^{2}} \end{matrix}$ (E.11)

so that if $\alpha(s)=\alpha$:

$V_{tot}^{q}(z,R,\omega)=\frac{1}{R^{3}}\frac{1}{\sqrt{2\pi}}\frac{\beta\alpha}{\text{v}}e^{-\frac{1}{2}\left(\frac{\beta\omega}{\text{v}}\right)^{2}}\int_{-\infty}^{\infty} e^{-i\omega\frac{s}{\text{v}}}\left\{\frac{2\frac{(z-s)^{2}}{R^{2}}-1}{\left(1+\frac{(z-s)}{R^{2}}^{2}\right)^{\frac{5}{2}}}\right\}ds$ (E.12)

Changing variables to:

$$y=\frac{\left(z-s\right)}{R}$$

it is now possible to write:

$\left|V_{tot}^{q}(z,R,\omega)\right|=\frac{1}{R^{2}}\frac{1}{\sqrt{2\pi}}\frac{\beta\alpha}{\text{v}}e^{-\frac{1}{2}\left(\frac{\beta\omega}{\text{v}}\right)^{2}}\left|g\left(-\frac{R}{\text{v}}\omega\right)\right|$ (E.13)

where:

$g\left(\omega\right)=\int_{-\infty}^{\infty} e^{-i\omega y}\left\{\frac{2y^{2}-1}{\left(1+y^{2}\right)^{\frac{5}{2}}}\right\}dy$ (E.14)

There are a number of important observations that should be made about Equation (E.13). First, the spectral response, as in many other problems, is the product of two factors, a “form factor” related to the spectral content of the impulse itself$\frac{1}{\sqrt{2\pi}}e^{-\frac{1}{2}\left(\frac{\beta\omega}{\text{v}}\right)^{2}}$ and a “structure factor” $\left|g\left(-\frac{R}{\text{v}}\omega\right)\right|$related to the distribution of the potential from a quadrupolar source. Considering first the “structure factor”, the integral in (E.14) can be carried out analytically using Mathematica (Wolfram Research, Champaign, Il) with the results shown in Figure 15. It is clear that g(x) decreases rapidly when , in fact, if G(x) is the cumulative power in the function g below$\omega$ :

$G(\omega)=\frac{\int_{0}^{\omega} \left|g\left(\omega'\right)\right|^{2}d\omega'}{\int_{0}^{\infty} \left|g\left(\omega'\right)\right|^{2}d\omega'}$ (E.15)

numerical integration reveals that 90% of the contribution of the “structure factor” to the total power in the recorded extracellular potential is for:

$$0<\frac{R}{\text{v}}\omega<3.37$$

It is clear that higher frequencies are recorded near the axon and only low frequencies far from the axon as in Table 2. On the other hand, contributions from the “form factor” are independent of R but do depend on the spatial extent of the action potential. Ninety percent of the power in the “form factor” occurs for:

$\omega<\frac{1.19\text{v}}{\beta}$ (E.16)

Taking $\beta=6\text{mm}$ and v=40meters/second, it is possible to tabulate the corresponding frequencies in Table 2. It is clear, as expected, that close to the axon, the highest recordable frequency is determined by the shape of the action potential but, at large distances, the highest frequency is more strongly determined by the distance from the axon. Figure 12 shows the power spectra expected from (E.13) as a function of distance from the axon.

**Appendix F: Radiation fields.**

Although electromagnetic radiation is not typically considered in clinical applications, changes in charge distributions over time produce radiation fields that typically decline in amplitude as$\frac{1}{R}$for large distances from the source and so could be significant when the recording electrodes are very far from the source. It is well known that charges moving at constant velocity in a uniform medium do not produce electromagnetic radiation unless the velocity is greater than the speed of light in that medium [7] and so true electromagnetic radiation can be produced from nerve impulses only when either the source charges are subject to acceleration, or when the polarization charges accelerate as the source charge moves through a region of altered dielectric constant, “Transition Radiation” [32,33]. It should be noted that even a fixed dipole that undergoes rigid linear acceleration does not generate any radiation to the lowest order approximation, except when it changes direction. A full discussion of radiation emitted in these situations is beyond the scope of this paper but it is simple to note that the classical formula for the power radiated by an accelerating dipole is [7,34].

where is the magnitude of the second derivative of the total dipole moment with respect to time. In MKS units the relation between potential and dipole density

$$\left|\vec{d}\right|=\varepsilon_{0}V_{m}$$

where:

$$\varepsilon_{0}=8.854x10^{-12}coul^{2}/newton-meter^{2}$$

and:

where $\omega$ is the angular frequency of the change describing the change in dipole moment over time. so that the total power will be on the order of:

$$I=\frac{1}{4\pi\varepsilon_{0}}\frac{2}{3c^{3}}\left(\pi\omega^{2}a^{2}\varepsilon_{0}V_{m}\right)^{2} (mks)$$

Substituting some typical values: V_m_=0.1Volt=0.1J/C=0.1 Nm/C, w=1000/sec a=1x10^-6^m it is possible to see that:

$$I\sim10^{-49}Watt$$

which is clearly tiny. Even if frequencies up to 100kHz are considered and the effect of 10^6^ synchronized axons were additive this figure would only increase to only 10^-35^ Watt which is still more than 10 orders of magnitude less than the power received from deep space probes at the edge of the solar system. The above calculations explicitly estimate only the radiation power produced by either the leading or trailing dipole, it is expected [31] that the total power emitted by the pair may be either of this order or smaller.

Frank [32] has discussed transition radiation and shown that the power radiated when a charge moves through a boundary between regions of differing dielectric constant is of the same order of magnitude although it exhibits a complex angular dependence and a complex dependence on the dielectric constants of the two media. A dipole moving through an interface may be expected to generate even less radiation.
